# The mechanical and microstructural characteristics of low-energy calcined clay from a high-Egyptian gray clay with hydrated lime for rendering mortar applications

**DOI:** 10.1038/s41598-026-37982-1

**Published:** 2026-02-27

**Authors:** Karam S. Salama, Essam A. Kishar, Doaa A. Ahmed, Mohamed E. Saraya, Ezzat A. El-Fadaly, Aya Allah M. Ebrahim

**Affiliations:** 1 Arab Swiss Engineering Company (ASEC), Cairo, Egypt; 2 Chemistry Department, Faculty of Women for Arts, Science and Education, Ain Shams University, Cairo, 11757 Egypt; 3 Department of Chemistry, Faculty of Science, Al-Azhar University, Cairo, Egypt; 4 Environmental Studies and Research Institute, University of Sadat City, Sadat City, Egypt

**Keywords:** Calcined clay, Hydrated lime, Render mortar, Inorganic pigment, Engineering, Environmental sciences, Materials science

## Abstract

The production of Portland cement has a significant global impact. Consequently, research into clinker replacement materials and alternative low-CO_2_ cements is becoming increasingly important to minimize their carbon footprint. For this study, limestone and raw clay were sourced from various locations in Egypt. These materials were calcined in a kiln at temperatures of 950 °C and 750 °C for 2 h, respectively. The primary goal of this study is to prepare and render mortars based on calcined clay with lime as a low-energy binder. The research primarily focuses on the mechanical and microstructural properties of lime-clay mortars and lime-clay render mortars, specifically hydrated lime (HL) and calcined clay (CCL). The mechanical and physical properties of these mortars were tested according to EN 196-1, using a mixing ratio of 1:3 (binder to sand) with total weight proportions of 450:1350. Water was added to achieve the necessary consistency and workability for this type of mortar, with an amount of approximately 302 ± 5 g. The results suggest that the most recommended proportion for optimal mortar performance with the used binder (CCL-HL) is L40, mainly consisting of 60% CCL and 40% HL with a water-to-solid ratio of approximately 0.17. The compressive and flexural strengths for the CCL-HL mortar sample (L40) are 13.2 MPa and 3.2 MPa, respectively. Additionally, the preparation of render mortars using this binder (L40) demonstrates that the most cost-effective and environmentally friendly binder proportion is R35Y, which consists of 35% L40 and 2% Fe_2_O_3_ as a yellow pigment, with a water-to-solid ratio of approximately 0.30. The compressive and flexural strengths of the R35Y render mortar are 5.2 and 2.1 MPa, respectively. The findings demonstrate that all prepared CCL-HL render mortars are suitable for rendering applications, meeting the requirements of EN998-1/2010 and offering a cost-effective, environmentally conscious solution for decorative and protective coatings, particularly in the preservation of historic structures.

## Introduction

Lime is highlighted as a sustainable alternative to Portland cement because of its lower energy use and CO_2_ emissions, as well as its ability to absorb CO_2_ through carbonation. Although Portland cement replaced lime in many applications due to its faster hardening, higher strength, and longer-lasting perception, Lime-based renders are often essential for compatibility in building conservation^1–3^. This is especially true when working with older structures that utilize lime mortars. The breathable characteristics of lime render allow moisture to evaporate, helping to prevent dampness and associated deterioration. However, applying lime render requires careful attention and a solid understanding of the existing substrate. If not applied correctly, lime render can fail prematurely, leading to further issues. Key factors such as aggregate grading, binder content, and curing conditions all significantly impact the long-term performance of the render^1–3^. Additionally, incorporating calcined clay into lime mortars enhances application speed, durability, and mechanical strength while ensuring compatibility with existing materials. Lime, which is derived from limestone, primarily serves as a mortar alternative to cement. Unlike the hydration process of cement, pure lime hardens and gains strength through carbonation.

Up to 5–8% of the world’s global CO_2_ emissions originate from the cement industry, which is facing increasing pressure to reduce its carbon footprint to meet carbon neutrality targets. Researchers are now developing alkali-activated concrete (AAC), a highly sustainable option that utilizes recovered powder (RP) from construction waste^4^. In the area of durable building materials, the creation of innovative binder materials by combining cement, phosphogypsum (PG), and ground granulated blast-furnace slag (GGBFS) has become essential^5^. These materials can incorporate more solid waste and produce fewer carbon emissions. Additionally, lightweight ultra-high performance concrete (L-UHPC), which has air-drying densities of approximately 1800 kg/m3 and compressive strengths exceeding 120 MPa, has emerged as a significant advancement in cementitious materials^6^.

This approach decreases natural clay use, energy consumption, and carbon dioxide emissions, benefiting the construction industry with low-carbon concrete and mortar options^7^. Additionally, the carbonation of calcium hydroxide Ca(OH)_2_ in an aqueous medium results in the formation of precipitated calcium carbonate (CC), which improves concrete durability^8^. Currently, researchers are exploring the use of additional industrial wastes and alternative supplementary materials, such as sand washing slurry (SWS) and granite residual soil (GRS). GRS contains a high amount of kaolinite, which is highly hydrophilic and easily disintegrates in water; it also reacts with an alkaline solution to form cementitious materials, enhancing the compressive strength of GRS^9–11^.

In order to supply enough calcium hydroxide needed for the pozzolanic reaction to increase the strength of the hardened material, hydrated lime can be added to the mixture that has a high percentage of Supplementary Cementitious Materials (SCMs)^12^. Using hydrated lime greatly speeds up the pozzolanic reaction, which helps preserve a denser microstructure and improve mechanical qualities. According to Jaafri et al.^13^, adding a high percentage of hydrated lime (12.5–50 weight percent of the binder) affected the fresh and hardened behaviors of mortars made with plain Portland cement and mortars blended with limestone. Lime mortar (LH) is one of the oldest types of mortar known and has been debated for its use in construction for the past 10,000 years, mainly in countries like India, Italy, Greece, and Egypt. Egyptians used air lime mortars as bedding mortars, while hydraulic lime mortar with pozzolans was extensively used by the Romans for improved strength and durability^7^.

Calcined clay (CCL), an artificial pozzolan created by heating certain clay types, improves the mechanical strength and water resistance of lime mortars, making them suitable for conservation. In contrast, cement mortars are not ideal for this purpose due to their brittleness, low plasticity, high elastic modulus, and soluble salts. Moreover, lime/pozzolan mortars have a smaller environmental footprint than cement mortars because they consume less energy during manufacturing and absorb CO_2_. Metakaolin, a specific kind of calcined clay, further enhances the durability of mortars and concrete^14^. Its application as a pozzolanic additive has become more popular recently^15^, especially in countries like the U.K. and the U.S., though its use remains limited in Portugal^16^. Metakaolin features an amorphous structure, a large surface area, and a high content of acidic oxides (primarily Al_2_O_3_ and SiO_2_, which make up over 90%), all of which facilitate its rapid reactivity and effective bonding with portlandite^17^. During calcination, the kaolinite structure breaks down, releasing –OH groups and forming an amorphous aluminosilicate (Al_2_O_3_·2SiO_2_), known as metakaolinite. The thermal transformation of kaolinite into metakaolinite generally involves three stages^18^. Initially, as heating starts, kaolinite’s structure decomposes, separating alumina and silicate layers and disrupting its orderly arrangement. This process continues until the temperature reaches 400 °C, driven by dehydroxylation of hydroxyl groups, which results in water evaporation. The breakdown occurs relatively early, between 100 and 200 °C, and persists up to 400 °C, alongside initial disintegration. During dehydroxylation, –OH groups split into H^+^ and O^2−^ ions. Protons combine with other -OH groups to produce water, which then evaporates. The O^2−^ ions remain within the metakaolinite crystal lattice, which reforms afterward. Around 600 °C, the alumina and silica layers reassemble to form metakaolinite (Al_2_Si_2_O_7_) with a crystalline structure similar to that of kaolinite^19^. The dehydroxylation process is represented as:

1 $${\mathrm{Al}}_{{2}} {\mathrm{Si}}_{{2}} {\mathrm{O}}_{{5}} \left( {{\mathrm{OH}}} \right)_{{4}} \to {\mathrm{Al}}_{{2}} {\mathrm{Si}}_{{2}} {\mathrm{O}}_{{7}} + {\mathrm{2H}}_{{2}} {\mathrm{O}}$$

At higher calcination temperatures (925–950 °C), metakaolinite transforms into an aluminum–silicon spinel structure (Si_3_Al_4_O_12_), expressed as follows:

2 $${\text{2 Al}}_{{2}} {\mathrm{Si}}_{{2}} {\mathrm{O}}_{{7}} \to {\mathrm{Si}}_{{3}} {\mathrm{Al}}_{{4}} {\mathrm{O}}_{{{12}}} + {\mathrm{SiO}}_{{2}}$$

Further calcination at ~ 1050 °C converts the spinel structure to mullite (3Al_2_O_3_·2SiO_2_), and heating at 1200 °C transforms the amorphous SiO_2_ into cristobalite SiO_2_:

3 $$3{\mathrm{Si}}_{{3}} {\mathrm{Al}}_{{4}} {\mathrm{O}}_{{{12}}} \to 2{\mathrm{Si}}_{{2}} {\mathrm{Al}}_{{6}} {\mathrm{O}}_{{{13}}} + 5{\mathrm{SiO}}_{{2}} \left( {{\mathrm{amorf}}} \right)$$

$${\mathrm{SiO}}_{{2}} \left( {{\mathrm{amorf}}} \right) \to {\mathrm{SiO}}_{{2}} \left( {{\mathrm{Cristobalite}}} \right).$$

The properties of metakaolin produced by calcination depend on both calcination time and temperature, with an ideal range of 550–800 °C, according to research^20^. Mechanical treatment reduces particle size and increases specific surface area. Milling causes gradual structural disordering, leading to kaolinite amorphization. This amorphous structure results in more pozzolanic metakaolin^21^. Clay materials with iron oxide turn red after heating, potentially discouraging commercial use as Sustainable Cement materials (SCMs) in the cement industry. However, due to their availability and low CO_2_ emissions, calcined clays offer a sustainable alternative to OPC, despite their non-renewable nature^22^.

The incorporation of inorganic pigments into mortar, while offering aesthetic benefits, presents a complex interplay of effects on the material’s workability and hardened properties^23,24^. Research^23^ indicates a clear relationship between pigment concentration and mortar fluidity, with yellow pigment, characterized by its needle-shaped particles, demonstrably reducing fluidity as its mixing ratio increases. Specifically, exceeding a 6% mixing ratio of yellow pigment can diminish mortar flow to 180 mm, a level that negatively impacts workability^23^. This observation necessitates careful consideration of pigment concentration to maintain desirable application properties. Furthermore, the hardened properties of mortar are also influenced by the addition of inorganic color pastes^24^. A recent study^24^ has revealed an inverse correlation between the content of inorganic color and both compressive and flexural strength. This suggests that a high concentration of inorganic color can compromise the structural integrity of the hardened mortar. However, the type of pigment plays a significant role in determining the extent of this impact^24^. Yellow inorganic color, at a 5% content, has been shown to enhance compressive strength^24^, even surpassing that of control groups without pigment. Conversely, green inorganic color exhibits the least effectiveness in improving compressive strength^24^. Additionally, both flexural and tensile strengths decline as the inorganic color paste content rises. However, these strengths may increase at later ages, potentially exceeding those of the control group^24^.

Cementitious materials, essential in modern construction, display a complex interaction between composition, workability, and mechanical properties. Recent studies highlight how specific admixtures and supplementary cementitious materials impact these features^25,26^. Research^25^ stresses the importance of slump flow in achieving shape stability and buildability, identifying an optimal range between 140 and 200 mm (ASTM C 1437). Hydroxypropyl methylcellulose (HPMC), a common admixture, notably affects setting times. While increasing HPMC dosage extends both initial and final setting times, the initial setting time shows greater sensitivity to HPMC levels^25^. Higher HPMC concentrations lead to increased porosity within the cementitious matrix. This elevated porosity, in turn, reduces both compressive and flexural strengths, illustrating a key trade-off in material design^25^. Additionally, replacing hydrated lime with calcined clay, as investigated by the Marvila et al. study^26^, adds another layer of complexity. The study indicates that as the replacement level of hydrated lime with calcined clay rises, more water must be added to maintain the same workability. This results in a higher water/cement ratio, which can adversely affect mechanical strength, since a higher overall water/cement ratio typically leads to lower strength of the cementitious materials.

Cementitious renders play a crucial role in the construction industry, providing protection and aesthetic appeal to buildings. A key performance characteristic of these renders is their adherence to the substrate, quantified by the adherence stress (fu). Generally, cementitious renders exhibit an adherence stress range of 0.3 to 0.5 MPa. Current requirements specify a minimum value of 0.3 MPa or cohesive failure, as outlined in various standards. Specifically, adherence requirements for mortars are as follows: fu ≥ 0.3 MPa or cohesive failure (LNEC report 427/05); fu ≥ VD (value declared by the manufacturer) with cohesive failure (EN 998-1: 2010); fu ≥ 0.3 MPa, with no individual values lower than 0.2 MPa (DTU 26.1: 1994 (NF P 15-201-2)); and fu ≥ 0.3 MPa for renders with painting or ceramic finishes (NBR 13528: 1995)^27^. Lastly, the prospects and applications of calcined clay pozzolan combined with hydraulic lime in the building industry have been investigated^28^. The results indicated that a binder composition comprising 60% lime and 40% CCL achieved the highest compressive strength of 0.89 MPa after 28 days of curing. It was found that all binder mixtures, except the 100% lime and 0% CCL mixture, could be used for various applications such as masonry, rendering, plastering, and pointing, with compressive strengths ranging from 0.4 to 2.5 MPa after the same curing period.

This research distinguishes itself from existing studies by investigating the feasibility of using low-cost calcined clay (CCL) as a substitute for hydrated lime (HL) in render mortars, rather than focusing on replacing ordinary Portland cement (OPC). The production of both gray and white cement requires heating at temperatures that can reach up to 1450 °C, making it energy-intensive. Furthermore, the cost of producing white cement is about three times that of ordinary Portland cement, primarily due to the additional energy required for firing and the use of fluxing agents such as CaF_2_. In contrast to OPC, CCL has a larger surface area and contributes significantly less to carbon dioxide (CO_2_) emissions; cement production releases approximately one ton of CO_2_ for every ton produced, while both hydrated lime and calcined clay have a lower environmental impact. At the same time, Egyptian gray clay, a valuable resource in Egypt, has specific characteristics that provide considerable utility. Its fine particle size and mineral composition make it suitable for various applications, including traditional pottery, brick-making, and innovative construction and environmental remediation uses. By understanding and utilizing the unique properties of Egyptian gray clay, we can maximize its potential, promoting sustainable development and showcasing its innovative applications across diverse fields.

This study presents a new calcined clay-hydrated lime binder that serves as a comprehensive and sustainable alternative to conventional cement or pure lime in render mortar formulations. Shifting away from traditional strength-focused assessments, the research emphasizes this binder’s suitability for specialized applications, particularly in enhancing the aesthetic qualities of buildings and aiding in the preservation of historical structures through its use in colourful ornamental renders.

**Fig. 1:**
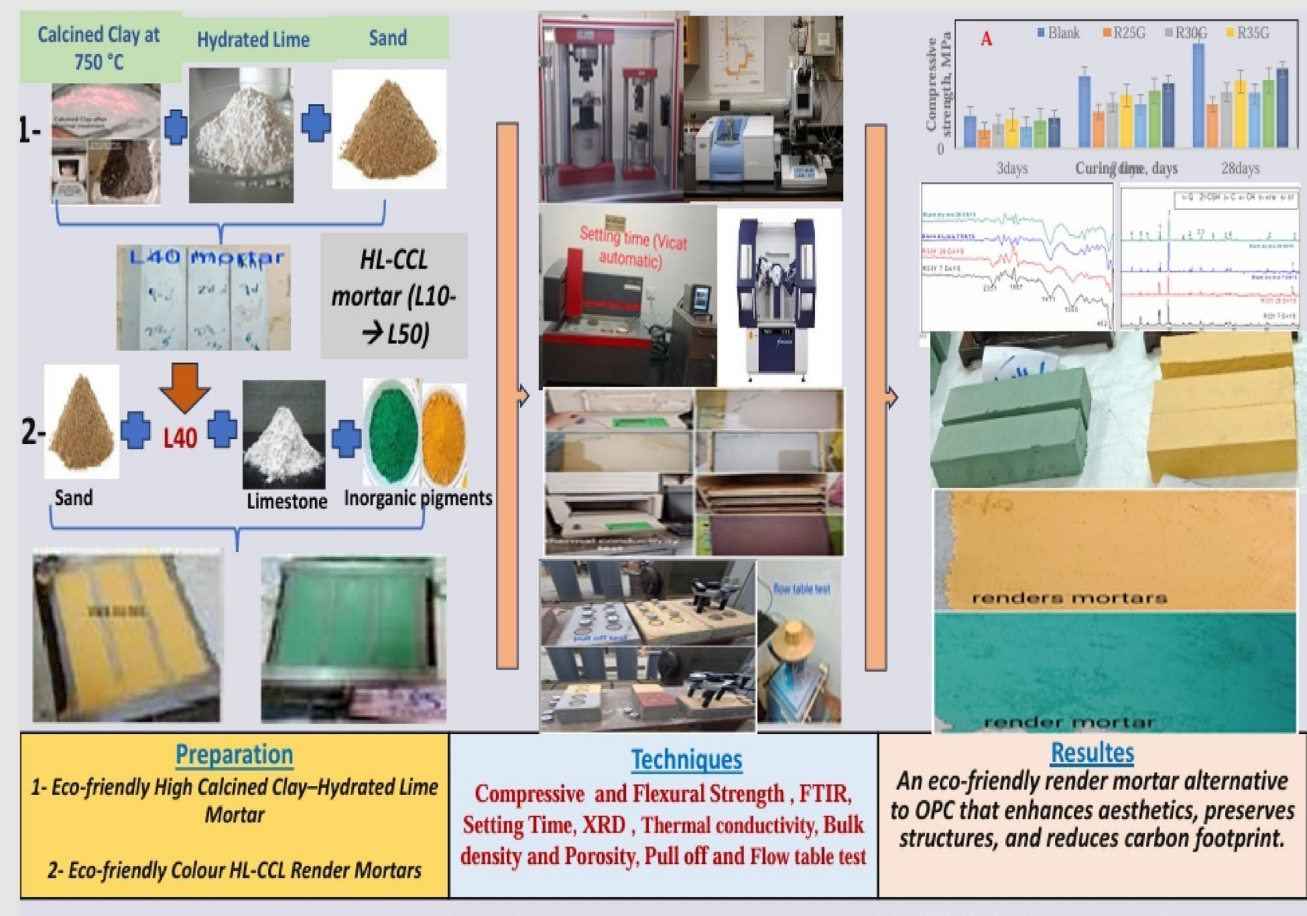
Schematic diagram illustrating the objectives of our study.

This method utilizes Egyptian gray clay, heated at 750° C, to produce a binder with lower production costs and reduced energy consumption. The experimental plan includes various CCL proportions—specifically 90%, 80%, 70%, 60%, and 50%—mixed with HL. The resulting CCL-HL mortars, made with Egyptian gray clay, are designed to function as low-energy binders in render mortars, reducing the environmental impact associated with Portland cement-based materials. In the second phase, the CCL-HL binder is tested as a colored render mortar for decorative purposes. To evaluate their performance, the mechanical and microstructural properties of these render mortars, which contain 1%, 1. 1.5%, and 2% inorganic pigments (green and yellow), are tested according to EN 998-1/2010 standards. The results show that these render mortars meet the EN 998-1/2010 standards for mechanical performance, indicating their potential for broad application. Additionally, the practical use of the CCL-HL binder as a colored render mortar for decorative applications is thoroughly examined, with the main goal of offering a sustainable alternative for rendering, enhancing building aesthetics, supporting the preservation of historic structures, and reducing the carbon footprint associated with traditional cement mortars—thus advancing the development of environmentally responsible construction materials (refer to Fig. 1).

## Materials and methods of investigation

### Materials

**Fig. 2:**
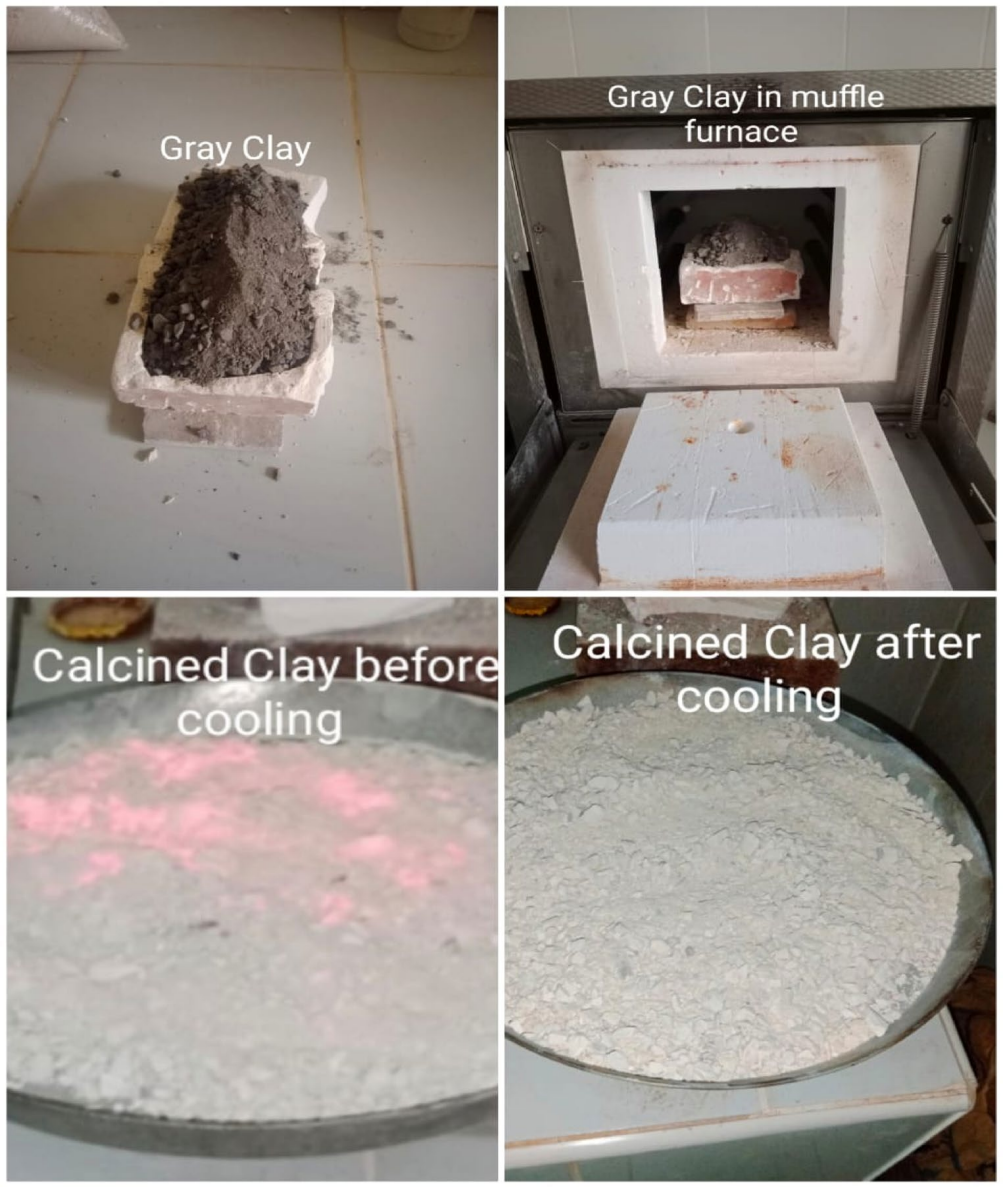
Calcination processes of gray clay.

The materials used in this study included HL from Heliopolis for a mining plant in El 10th of Ramadan, Sharqia, Egypt, and CCL, which was prepared in the laboratory as shown in Fig. 1. The gray clay was sourced from Ras Abu Zneima Zone, south of Sinai, Egypt. All the material such as white cement, Hydroxypropyl Methylcellulose (HPMC), limestone (LS), sand, and inorganic pigments (green as chromium (III) oxide and yellow as ferric oxide), which act as fillers in the preparation of dry renders were provided by Dry Mix Company, El 10th of Ramadan, Sharqia, Egypt. Figure 2a presents the particle size distribution of the initial raw materials. Hydrated lime is notably finer than the other materials, with most of its particles measuring between 0.01 and 0.04 mm. In comparison, the particles of sand, as illustrated in Fig. 2b, typically range from 0.2 to 1 mm. The chemical composition of the raw materials used in this study was analyzed using X-ray fluorescence (XRF), as detailed in Table 1.

**Table 1:** X-ray fluorescence (XRF) analysis of raw materials used in this study.

Oxides	SiO_2_	Al_2_O_3_	Fe_2_O_3_	CaO	MgO	SO_3_	Na_2_O	K_2_O	TiO_2_	P_2_O_5_	CL	LOI	Sum
HL	0.14	0.11	0.05	64.72	0.74	0.09	0.00	0.00	0.00	0.08	0.01	34.00	99.86
Gray Clay	53.38	30.80	3.74	0.02	0.15	0.12	0.13	0.11	1.91	0.04	0.03	9.58	99.96
Calcined clay	55.65	34.20	5.24	0.23	0.01	0.15	0.15	0.13	2.55	0.11	0.07	1.50	99.92
Limestone	0.38	0.08	0.02	54.40	0.96	0.07	0.21	0.25	0.00	0.00	0.02	43.60	99.99
Sand	96.33	1.20	0.53	0.09	0.10	0.05	0.12	0.51	0.00	0.00	0.01	0.95	99.89
White cement	20.65	4.95	0.43	64.88	1.08	2.45	0.10	0.56	0.42	0.02	0.04	4.20	99.75

#### Preparation of calcined clay (CCL)

**Fig. 3:**
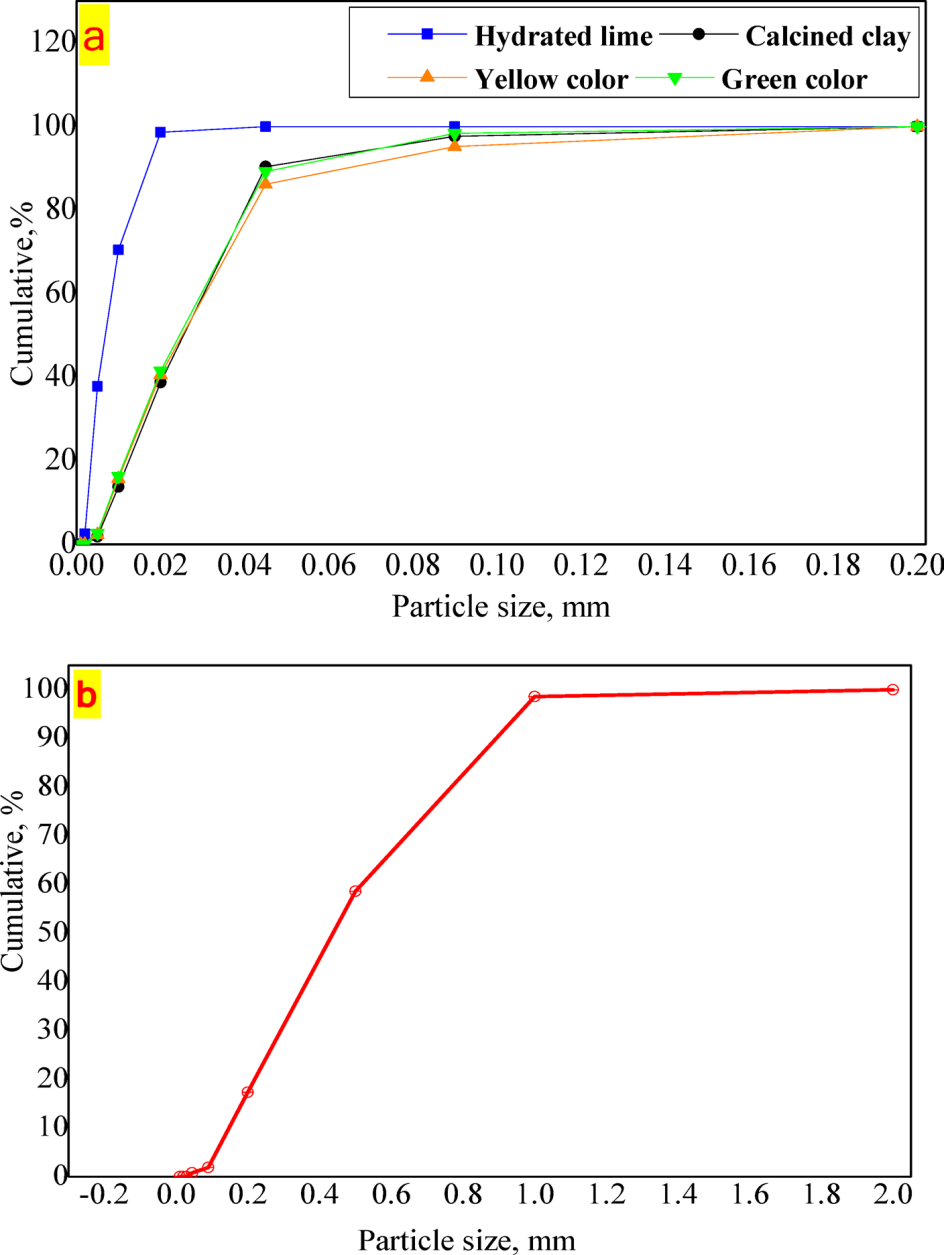
Particle size distribution for: (**a**) the initial raw materials, and (**b**) the sand.

Figure 2 shows the process of preparing calcined clay, which is a key material for making three different binders. During calcination, raw clay is dried at 120 °C to remove moisture, then ground and sieved into a fine, uniform powder (1 mm). The calcination of raw clay was carried out in an electric muffle furnace (Model: Nabertherm L 5/12, Germany; maximum temperature 1200 °C), equipped with a digital PID controller and K-type thermocouple to ensure accurate temperature regulation. The heating rate was set to 6 °C/min, and calcination was conducted at 750 °C with a holding time of 2 h. This heating causes a visible color change from gray to light red, due to iron oxide in the clay. After thermal treatment, and in the absence of oxygen, magnetite converts into hematite, resulting in a light red colour for the material. If oxygen is present during cooling, magnetite may revert to hematite, producing a reddish hue. Without oxygen, magnetite remains the stable phase^22^. The calcined clay was then finely ground in a small laboratory ball mill with a capacity of five kilograms. Figure 3a shows the particle size distribution of the ground calcined clay; after sieving through a 45-micron mesh, about 10% of the material was left as residue.

#### Mineralogical and microstructure characterization

**Fig. 4:**
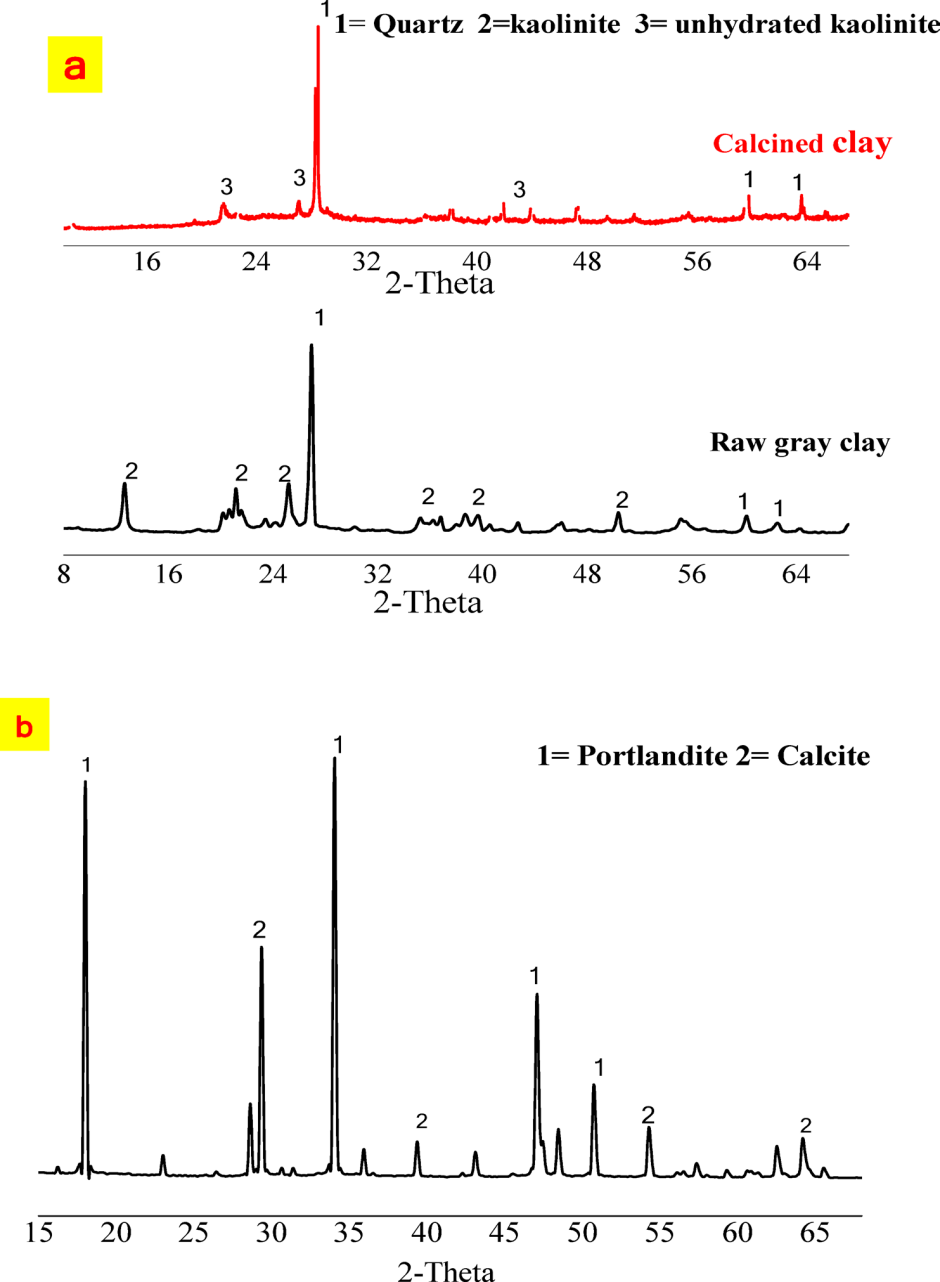
X-ray Diffraction (XRD) patterns of: **a** Kaolinite clay and calcined clay, **b** Hydrated lime.

The mineralogical composition of clay before thermal treatment, as well as that of calcined clay and hydrated lime, was analyzed using X-ray diffraction (XRD) and Fourier-transform infrared spectroscopy (FTIR). Figure 4a shows the presence of quartz, unhydrated kaolinite, and the most reactive component, which is the amorphous aluminosilicate phase. After calcination, the high-intensity sharp edges in the raw clay, especially those at 2θ = 12°, 20°, and 25°, disappear. The increased baseline between the major peaks indicates a broad, moderate hump in the low-angle region, specifically between 15° and 35° (2θ). This wide hump confirms the presence of an amorphous aluminosilicate phase (Al_2_Si_2_O_7_ or metakaolin), which is the desired byproduct of calcination. These amorphous phases form through reactions between the primary components, SiO_2_ and Al_2_O_3_^29^. Quartz peaks at 2θ values of 20° and 26° (d = 3.34 Å) were observed in both the clay and calcined clay^29,30^. Finally, the crystalline phases of calcined clay are quartz and unhydrated kaolinite. The most reactive component in calcined clay is metakaolin, an amorphous phase^31^. The XRD pattern of hydrated lime (Fig. 4b) shows a major mineral phase of calcium hydroxide at 2θ = 34.10° and 49.20° (d = 2.63 Å and 1.93 Å), along with a minor phase of calcium carbonate at 2θ = 29.40° and 39.40° (d = 3.03 Å and 2.28 Å). Calcium carbonate forms due to moisture on the lime’s surface reacting with atmospheric carbon dioxide, leading to the formation of thick granules of CaCO_3_.

**Fig. 5:**
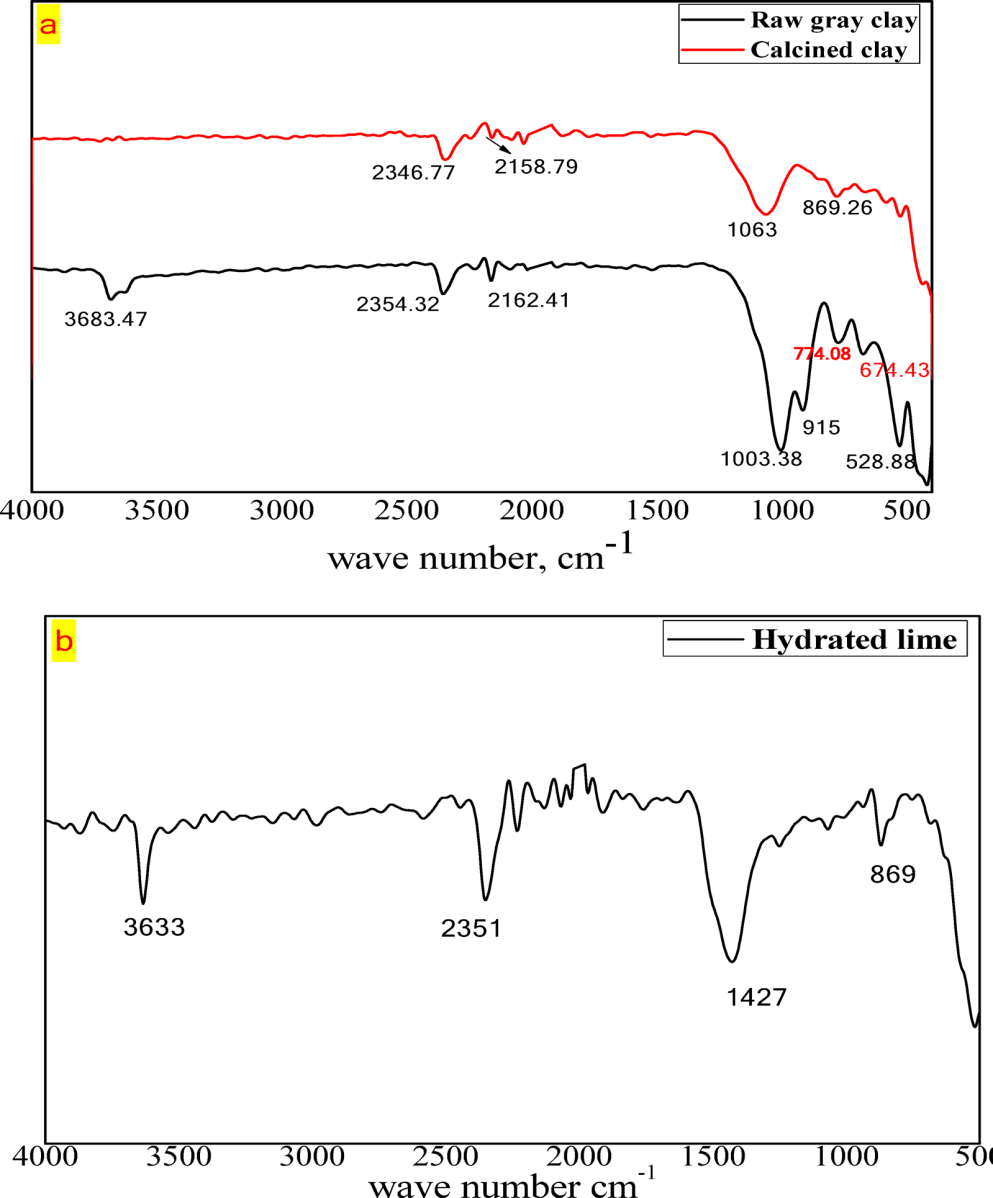
FTIR spectra of: (**a**) Raw gray clay and calcined clay, (**b**) Hydrated lime.

FT-IR spectra of clay before and after calcination are presented in Fig. 5a. The spectra of raw clay exhibit a strong peak at 3683 cm^−1^, which is attributed to the stretching of inner-surface (OH^−^) groups linking the clay sheets^19,29^. Additionally, a sharp peak at 915 cm^−1^, characteristic of Al–OH octahedral stretching vibrations, is observed in the spectra of clay before dehydroxylation. Strong peaks at 1003 cm^−1^ and 1063 cm^−1^ are also evident, corresponding to the stretching vibrations of Si–O bonds. The bands at 774 cm^−1^, 779 cm^−1^, and 674 cm^−1^ are assigned to the stretching vibrations of Si–O present in quartz. Finally, the bands at 532 cm^−1^ and 430 cm^−1^ are associated with Si–O Al VI bending vibrations and Si–O bending vibrations, respectively. In calcined clay spectra, the disappearance of the bands at 915 cm^−1^ and 3683 cm^−1^ indicates the successful completion of the dehydroxylation process. Moreover, a strong peak at 1063 cm^−1^ appears, attributed to amorphous silica. The decreased intensity and absence of the bands at 779 cm^−1^ and 674 cm^−1^ (related to quartz) indicate that these features remain in the spectra of calcined clay and are unaffected by thermal treatment^20,29^. Additionally, the absorption peaks at 2354 cm^−1^ and 2162 cm^−1^ in both calcined clay and kaolinite clay are due to CO_2_ vibrations adsorbed on the surface of the clay particles^21^.

Figure 5b displays the FTIR spectra of hydrated lime, showing characteristic bands of calcite at 873 cm^−1^. The strong band at 1423 cm^−1^, along with a weaker band at 2350 cm^−1^, corresponds to CO_3_^2−^ ions. The prominent O–H stretching band at 3640 cm^−1^ is due to the presence of Ca(OH)_2_, which is found only in lime hydrate^8^.

#### Preparation of CCL-HL mortars and CCL-HL colored render mortars

Different mortar mixtures made with high-content CCL and HL were prepared, as detailed in Table 2. Table 3 presents various render mortar mixes, including a blank sample, which was prepared using white cement and yellow pigment. Tables 1 and 4 show the chemical composition and physical properties of the white cement used.

**Table 2:** Mix design for the different prepared CCL-HL mortar mixes.

CCL-HL mortar mixes					
Sample No	HL (gm)	CCL (gm)	Sand (gm)	Water (ml)	W/S
L10	45.00	405.00	1350	307	0.171
L20	90.00	360.00	1350	305	0.169
L30	135.00	315.00	1350	303	0.168
L40	180.00	270.00	1350	302	0.168
L50	225.00	225.00	1350	298	0.166

**Table 3:** Mix design for the prepared render mortar mixes.

Render mortars based on L40 CCL-HL binder						
Mix. NO	White OPC (%)	L40 (%)	Sand (%)	LS (%)	In. pig (%)	HPMC (%)
Blank (Y)	30.00	–	52.90	15.00	2.00	0.10
R25G	–	25.00	58.90	15.00	1.00	0.10
R30G	–	30.00	53.40	15.00	1.50	0.10
R35G	–	35.00	47.90	15.00	2.00	0.10
R25Y	–	25.00	58.90	15.00	1.00	0.10
R30Y	–	30.00	53.40	15.00	1.50	0.10
R35Y	–	35.00	47.90	15.00	2.00	0.10

**Table 4:** Physical properties of white cement.

Blaine (cm^2^/g)	Whiteness degree (%)	Compressive strength (MP)	
		2 days	28 days
5200	93.00	21.80	48.60

### Test methods

#### Mixing and curing process

**Fig. 6:**
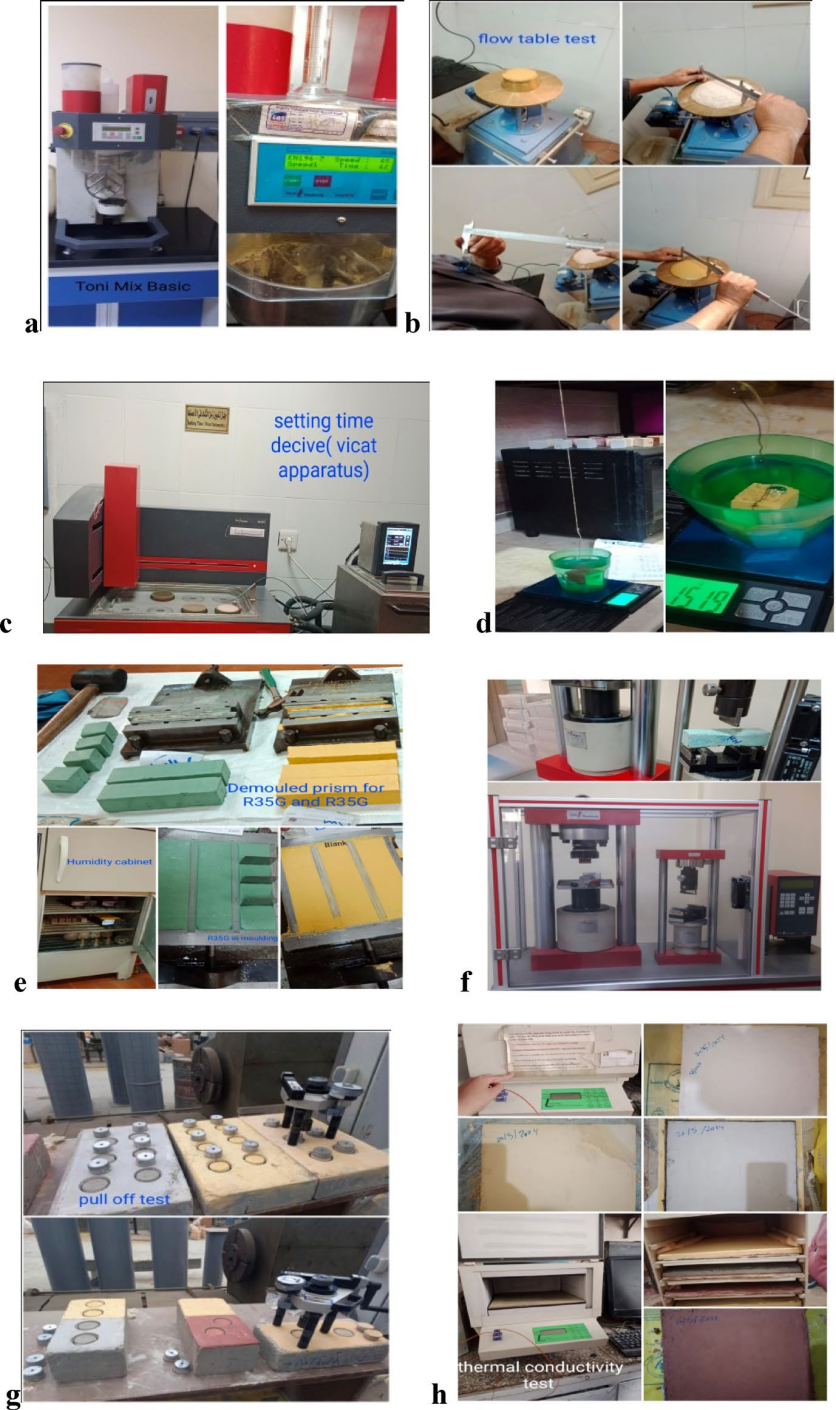
Test instrument used to prepare and investigate mechanical characteristics for prepared mortar mixes and render mortars: (**a**) Mortar mixer Toni MIX basic, (**b**) Flow table test, (**c**) Automatic vacate apparatus, (**d**) bulk density and porosity test, (**e**) The mold for creating mortar prisms, (**f**) Compression and bending test Plant Toni PRAX, (**g**) Pull off test, (**h**) Thermal conductivity test.

Mortars were prepared according to the proportions listed in Tables 2 and 3. The water amount was calculated to ensure proper flowability and workability for these mortars, achieving a flow value of about 150–185 mm after 15 jolts on the flow table. The mortar was mixed following the procedure in EN 196-1, with a fixed ratio of 450:1350. Water was added during the first seconds of mixing, which continued mechanically for 150 s (Fig. 6a), using a laboratory planetary mortar mixer (model Toni Mix Basic Mixer TD200, Toni Technik, Germany). The sides of the mixing container were scraped, and mixing was continued for an additional 30 s. The mortars were then cast into metallic prismatic molds measuring 40 mm × 40 mm × 160 mm. After demolding, the samples were cured in a constant temperature and humidity curing cabinet (model YH-40B, Cangzhou Zhongya Laboratory Instrument Co., China) at 90 ± 5% relative humidity and 20 ± 3 °C for 28 and 90 days. The day before testing, the samples were kept in a climatic chamber at 20 ± 3 °C and 65 ± 5% relative humidity for 24 h. Figure 6a–f presents all the test methods examined in this study.

#### Determination of consistency of fresh mortar using the flow table

The flow value is determined by measuring the mean diameter of a test sample by using a motorized flow table (model JJ-5, Cangzhou Zhongya Laboratory Instrument CO., China). The test procedure involves placing the mold (60 mm in height, with an internal diameter of 100 mm at the base and 70 mm at the top) in the center of the flow table (Fig. 6b). The mold is filled in two layers, with each layer tamped ten times using a tamper, as depicted in Fig. 6b. It is important to hold the mold firmly in place during this operation. Any excess mortar should be removed from the top of the mold using a palette knife, and the area around the base of the mold should be cleaned. After allowing approximately 15 s to elapse, the mold is removed. The table is then jolted 15 times at a rate of one jolt per second. The diameter of the spread mortar is measured in two perpendicular directions using calipers, and both measurements are reported. There are three states of flow table values as follows:

- Flow value less than 140 mm (stiff mortar)—Vibration method
- Flow value between 140 and 200 mm (Plastic mortar)—Shock method
- Flow value greater than 200 mm (Soft mortar).

#### Determination of setting time of fresh mortar by using the automatic Vicat needle

To determine the initial and final setting times of lime-based mortars, measure the time taken for the mix to lose plasticity and begin to harden using an automatic Vicat apparatus (model NZL-3, Cangzhou Zhongya Laboratory Instrument Co., China). Prepare the fresh mortars in the same mixer as specified in EN 196-1. Place water in the mix bowl, then add the lime-based mortars as shown in Fig. 6a–c; start the mixer at low speed (140 ± 5 rpm) for 30 s, then add sand gradually over 30 s, stop for 15 s and scrape down the bowl sides, then run at high speed (285 ± 10 rpm) for 60 s. Fill the Vicat mould (d = 70 mm, h = 40 mm) with fresh mortar, then place it under the Vicat apparatus and cure at 20 ± 2 °C and a relative humidity (RH) of 90 ± 5%. Take penetration readings every 30–60 min: the initial setting time is when the needle penetrates to 5 ± 1 mm from the bottom, and the final setting time is when the needle fails to make a visible mark on the surface of the mortar.

#### Bulk density measurements for hardened mortars

Bulk density (ρ-b) measurements were conducted before compressive strength testing. The specimens were first saturated and then weighed in both air and water (saturated surface dry condition), as illustrated in Fig. 6d. Each measurement was performed on at least three identical cubes with the same mix composition and curing age. The density was calculated using Archimedes’ method, following the formulas below:

$${\mathrm{Bulk}}\;{\mathrm{density}}(\uprho {\mathrm{-b}}) = \frac{{{\mathrm{Saturated}}\;{\mathrm{weight}}}}{{{\mathrm{Volume}}\;{\mathrm{of}}\;{\mathrm{sample}}}}\left( {{\mathrm{g}}/{\mathrm{cm}}^{3} } \right)$$

$${\mathrm{Volume}}\;{\mathrm{sample}} = \frac{{{\mathrm{Saturated}}\;{\mathrm{weight}} - {\mathrm{suspended}}\;{\mathrm{weight}}}}{{\left( {\uprho _{{\mathrm{w}}} } \right)}}\left( {{\mathrm{cm}}^{3} } \right)$$

$${\mathrm{Bulk}}\;{\mathrm{density}}\;\uprho {\mathrm{-b}} = \frac{{{\mathrm{Saturated}}\;{\mathrm{weight}}}}{{{\mathrm{Saturated}}\;{\mathrm{weight}} - {\mathrm{suspended}}\;{\mathrm{weight}}}}\left( {{\mathrm{g}}/{\mathrm{cm}}^{3} } \right)$$

where ρ_w_ is the density of water (≈ 1.0 g/cm^3^ at 20 °C); Saturated weight is the mass of the specimen in air after saturation; Suspended weight is the mass of the specimen while submerged in water.

#### Porosity measurement using EN 1936-2018, Archimedes (immersion method).

The apparent porosity (P%) of the mortar cubes (25 × 25 × 25 mm) was determined by three precise mass measurements. First, specimens cured under standard conditions are dried at 70 ± 5 °C for 24 h until a constant dried mass (Wd) is achieved, followed by cooling in a desiccator. Next, full saturation was achieved by immersion in boiled water for 2 h, and the saturated mass in air (Ws) is measured after removing only superficial moisture with a damp cloth. Finally, the saturated suspended mass (W_sus_) was recorded as shown in Fig. 6d, ensuring no air bubbles appear or adhere to the specimens’ surface or wire. The percentage of porosity is then calculated using the mass differential relationship:

$${\mathrm{P}}\% = \left[ {\left( {{\mathrm{Ws}} - {\mathrm{Wd}}} \right)/\left( {{\mathrm{Ws}} - {\mathrm{W}}_{{{\mathrm{sus}}}} } \right)} \right] \times {1}00.$$

According to EN 1936-2018, it is recommended to dry the specimens at 70 ± 5 not 105 ± 5 °C, as lime-pozzolana mortars contain chemically bound water and weak hydrates (CSH, CAH), which may dehydrate above 80 °C and change in their microstructure, so during immersion, we must not use hot water. Also, it is recommended to maintain a constant temperature of 20 ± 2 °C during weighing.

#### Compressive and flexural strength determination

Both compressive strength and flexural strength were determined according to EN 196-1 using prismatic test specimens measuring 40 mm × 40 mm × 160 mm. The mortar samples were composed of one part binder to three parts standard sand, with a water-to-binder ratio that ensured good workability. The mortar was prepared through mechanical mixing and compacted in molds using a standard jolting apparatus, as shown in Fig. 6e. The specimens in the molds were stored in a moist atmosphere for 24 h, after which they were demolded and stored at a relative humidity (RH) of 90 ± 5% and a temperature of 20 ± 3 °C. They were cured for up to 28 and 90 days. A day before testing, the samples were placed in a climatic chamber at 20 ± 3 °C and 65 ± 5% RH for 24 h. The test was applied to three samples for each mix. During the flexural test, each specimen was broken into two halves, and each half was subsequently tested for compressive strength, and then the average of the three was calculated. The compressive and flexural strength tests were conducted using the Automatic Compression and Bend test plant** (**model Toni PRAX Germany), as shown in Fig. 6f. Data collection was facilitated by a computer connected to a Zwic test Xpert system.

#### Adherence to the substrate (pull-off test)

Adherence is a property that enables a render to withstand both normal and tangential stresses at the interface with the substrate, even after the hardening process. Typically, a test involves using a digital or manual pull-off adhesion testing machine with a dolly by using a pull-off Adhesion Tester (model Proceq DY-216 Elcometer506(UK), with a capacity up to 20MPa, according to BS1881-207:1992. Before beginning the test, it is essential to clean both the steel dolly (50mm) and the coated surface. Next, prepare the adhesive and apply it to the dolly, which is then attached to the coated surface after 28 days. Allow the adhesive to cure at room temperature. Once the curing process is complete, the machine’s actuator is positioned over the steel dolly, which is attached to the specimen’s surface by epoxy resin, and the load is applied perpendicularly at a controlled rate of 0.05 ± 0.02MPa until the bond fails. This pressure can be generated using a hydraulic pump, as illustrated in Fig. 6g, and the bond strength was calculated by dividing the maximum load by the dolly area.

#### Thermal conductivity test

The amount of energy required for the heating and cooling of buildings is dependent on the thermal conductivity (k-value). Conduction heat transfer in solids occurs through molecular vibrations and energy transport by free electrons. The thermal conductivity of the present mortars in this study is determined by using a laser comp heat flow meter Fox 314 instrument with S/N: 11061327, which was performed at the thermal lab at Housing and Building National Research Centre, HBRC. According to ASTM C518, which is a standard test method for determining steady-state thermal transmission properties of materials using a heat flow meter apparatus. This method measures the thermal conductivity and thermal resistance of materials like insulation and construction materials. The test involves placing a specimen with certain dimensions (30 × 30 cm × 1 cm) between two plates, one heated and the other cooled, and measuring the heat flow through it as illustrated in Fig. 6h. The plates are maintained at different temperatures, creating a controlled temperature difference (temperature gradient) across the specimen, which was 25 °C, and the mean temperature difference across the specimen is 32.5 to 45 °C. The heat flux direction through the sample was upwards from the hot plate to the cold plate for 5 h in environmental conditions during the test, which were (20 °C and RH = 55%).

## Results and discussion

### CCL-HL as binder in mortars

Pozzolanic materials CCL can be combined with uncarbonated lime HL to create stable compounds, which reduces the risk of early leaching or frost damage. This combination also improves the mortar’s mechanical properties and potential durability^7^. Different mixes were prepared according to the percentages outlined in Table 2 to assess the effect of a high content of natural pozzolana (CCL) on HL mortar mixes.

#### Water demand and setting time

**Fig. 7:**
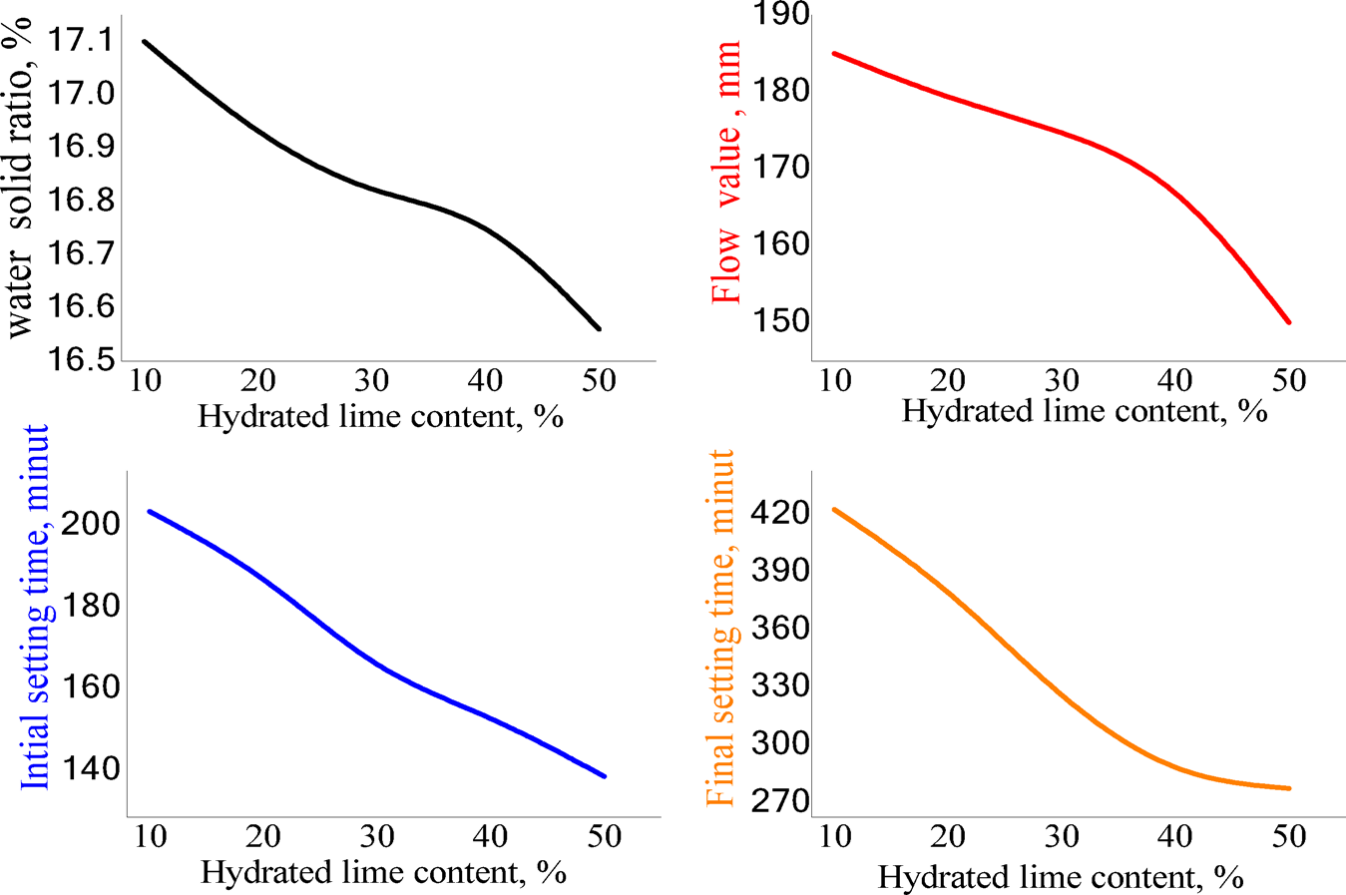
Different characteristics of HL-CCL mortars, including water consistency, flow value, initial and final setting time.

The results indicated that the water demand of the mortars is influenced by the lime-to-pozzolana ratio in the mortar mixes, as illustrated graphically in Fig. 7. There is a clear relationship between water demand and HL content; specifically, replacing pozzolana with HL reduces both water demand and the setting time of all CCL-HL mortar mixes (L10-L50). As the amount of HL increases and the amount of CCL decreases, both water demand and setting time decrease due to the enhancement of the pozzolanic reaction, which proceeds at a slower rate than the carbonation reaction^21^. However, after 22 h, the rate of the pozzolanic reaction surpasses that of the carbonation process in the mortars. Mixes with higher amounts of CCL tend to have a greater water demand^21^, which may be attributed to the lower plasticity and higher specific surface area of the CCL. Consequently, mortars containing larger quantities of CCL require more water to maintain a consistent index^26^.

The water demand of the mortars is strongly influenced by the relative proportions of hydrated lime (HL) and calcined clay (CCL). Increasing the CCL content leads to higher water demand due to its higher specific surface area, angular particle shape, and porous microstructure, which promote greater water absorption and reduce mixture fluidity^32^. In contrast, hydrated lime consists of finer, plate-like particles that improve lubrication, enhance plasticity, and increase water retention, thereby reducing the amount of water required to achieve a workable consistency^33^. Consequently, mixtures with higher HL/CCL ratios require lower water content, while increasing the proportion of CCL necessitates additional water to maintain similar workability. However, since the sand content is kept constant, the overall variation in water demand among the mixes remains moderate, as the water-absorbing effect of CCL is partially offset by the plasticizing and water-retaining characteristics of HL^34,35^.

#### Mechanical properties of CCL-HL mortars

**Fig. 8:**
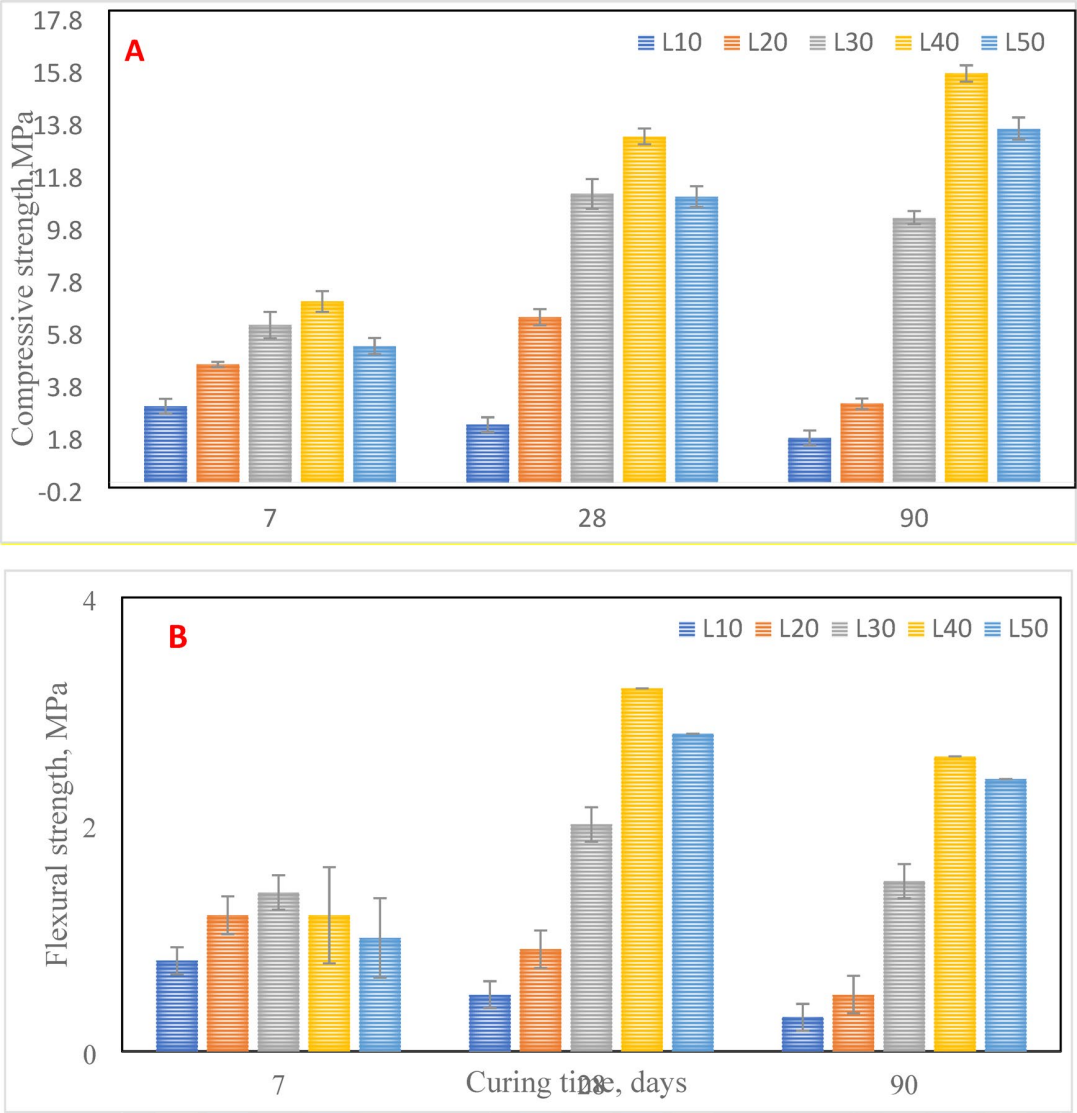
Mechanical properties of different CCL-HL mortars under different hydration ages, (**a**) Compressive strength, (**b**) Flexural strength.

The compressive and flexural strength tests were conducted according to standard EN 196–1. The results, shown in Fig. 8a, b, indicate that the compressive strength increases over time, reaching its maximum at 90 days for mortar samples with 40% and 50% replacement of CCL with lime (L40 and L50). This improvement is attributed to enhanced pozzolanic reactions and the formation of additional hydration products. In these mixes, the reaction can be summarized as follows: Calcium Hydroxide + CCL → Calcium Silicate Hydrate (CSH). The CSH phase is the main contributor to strength in concrete and cementitious materials^36^. The pozzolanic reaction mainly occurs at shorter curing times (up to 28 days), while carbonation becomes more significant at longer curing periods. As previously mentioned, stratlingite plays a crucial role in improving the mechanical strength of CCL-HL mortars, which increases with both ageing and CCL content^19,37^. This phenomenon has also been observed in other mortars containing pozzolans. Although the reasons for these effects are still unclear, they may be linked to microcracking caused by shrinkage, which is particularly sensitive to flexural strength^38^. Higher lime content, up to 40%, boosts both compressive and flexural strength at early ages, as shown in Fig. 8a, b. This improvement is due to the interaction between HL and CCL as a binder, which promotes the production of hydration products. Overall, both compressive and flexural strength tend to increase as the percentage of HL added rises, except for the L50 mix at 7 days. A 40% HL addition (L40 mix) appears to be optimal for the CCL-HL mortars. The reduction in mechanical resistance seen in some mortar mixes from 28 to 90 days relates to the instability of calcium aluminate hydrate in the presence of lime^37^. However, this instability is expected to resolve once the high lime content is fully consumed through either pozzolanic or carbonation reactions.

Figure 8 presents the standard deviation (SD) values for the results, which indicate good reproducibility. The lowest SD values were observed in L10 and L20, especially at early ages, suggesting more uniform hydration. In contrast, the slightly higher SD values for L30 to L50 indicate heterogeneous pozzolanic reactions and local variations in the matrix. After 90 days, the SD values generally stabilized, reflecting improvements in microstructural uniformity. These findings are consistent with the reports from Khilfi and Chappo^39^ and Chen et al.^40^, who noted minor variability in mortars containing higher pozzolan content.

#### XRD analysis

**Fig. 9:**
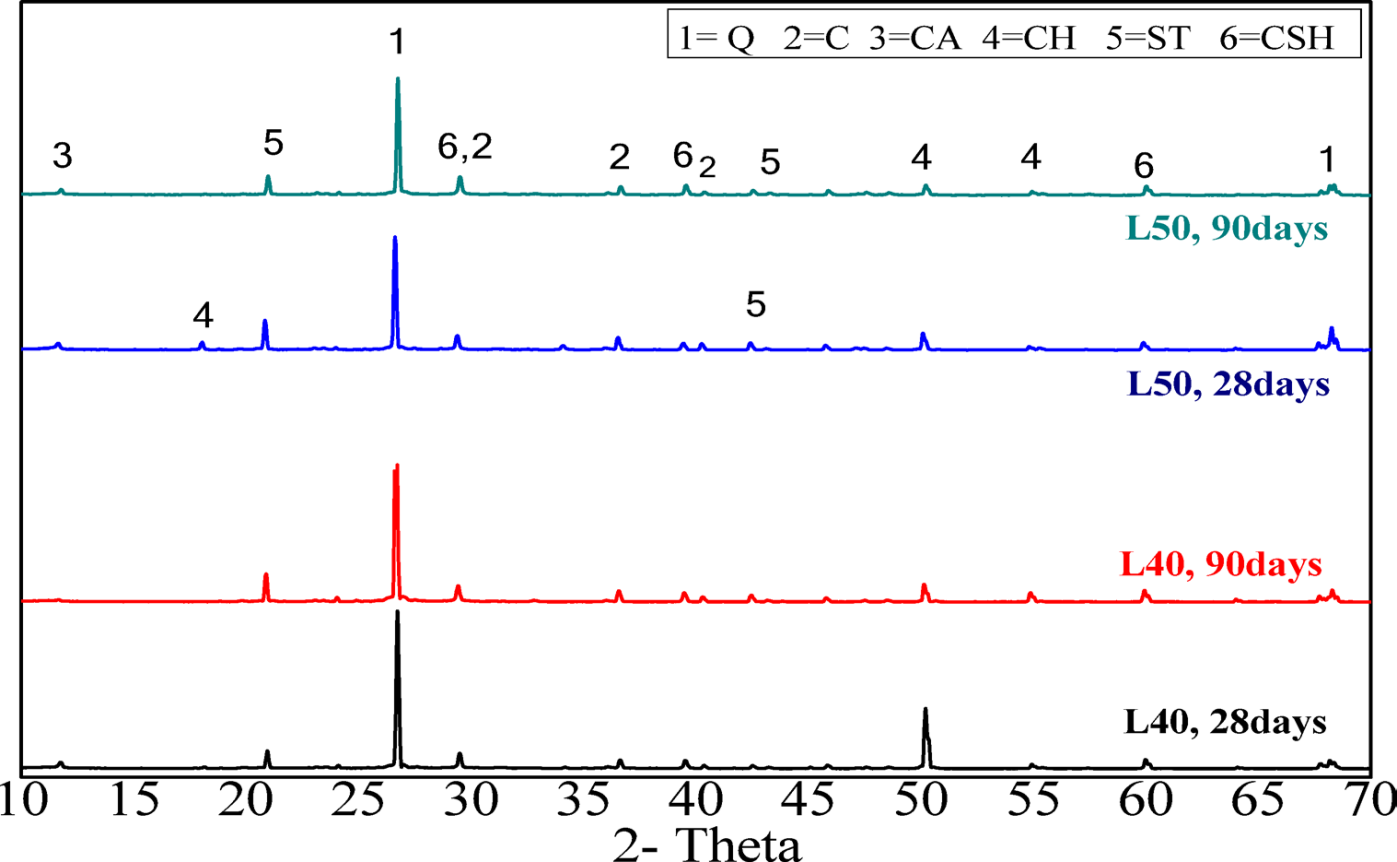
XRD patterns of CCL-HL mortar mixes (L40, L50) after 28 and 90 days of hydration (Q = quartz, C = calcite, CA = calcuim carboaluminate, CH = portlandite, ST = stratlingite, CSH = calcium silicate hydrate).

**Fig. 10:**
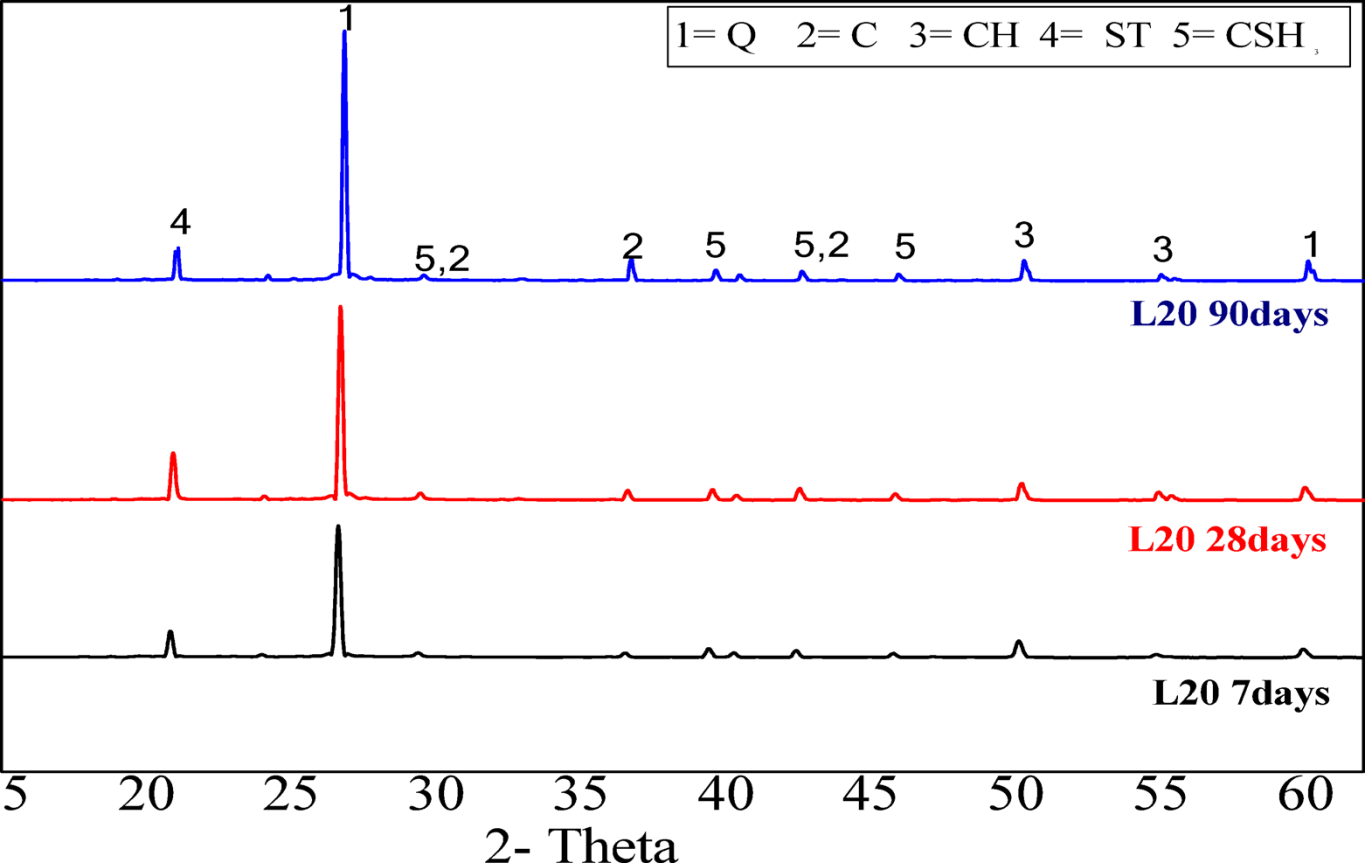
XRD pattern of CCL-HL mortar mix (L20) after 7, 28, and 90 days of hydration. (Q = quartz, C = calcite, CH = portlandite, ST = Stratlingite, CSH = calcium silicate hydrate).

Figure 9 presents the XRD pattern for the L40 and L50 mortar mixes at 28 and 90 days of curing. It is observed that the main phases of the hydrated mortars include stratlingite (C_2_ASH_8_ with d values 4.18, 2.87, 2.49 Å), calcium silicate hydrate (CSH with d-values 3.06, 2.78, 1.8, and 1.6 Å), Calcium carboaluminate ( CA with d values 7.88, 2.82 Å), portlandite (CH with d values 4.8, 2.69, 1.93 Å), quartz Q with d values 3.34 2.69 Å, , and calcite due to carbonation, with d values 3.03, 2.76 Å. The intensities of the peaks increase with curing time up to 90 days, attributed to enhanced pozzolanic reactions^41^. Conversely, the intensity of the CH peak decreases over time, which is also a result of the pozzolanic reaction. Furthermore, the presence of CCL and its associated high pozzolanic reactivity leads to lower levels of dissolved portlandite^29,41^. Figure 10 illustrates the XRD pattern of the L20 binder mix, which exhibits the same phases as the previous mixes. However, a reduction in the stratlingite peak for the L20 mix at 90 days is noted, correlating with a decrease in mechanical strength, which is in good agreement with compressive strength results (Fig. 8). A key observation from these Figures is the presence of calcium aluminate^42^.

#### Fourier transform infrared spectroscopy (FTIR)

**Fig. 11:**
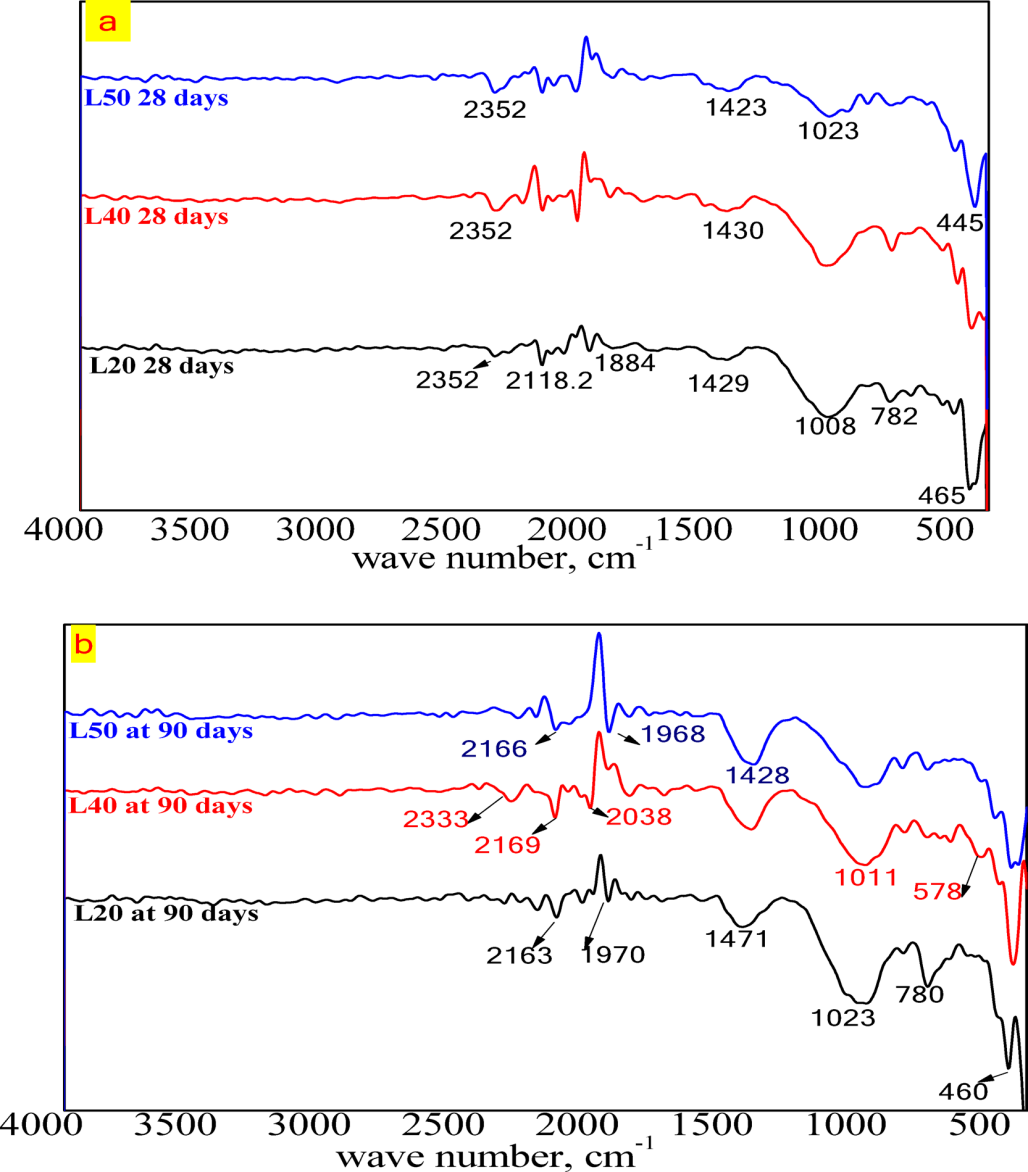
FTIR patterns of CCL-HL mortar mixes L20, L40, and L50 at: (**a**) 28 days, and (**b**) 90 days.

FTIR spectra can be used to analyze the changes in functional group environments in CCL-HL mortar mixes (e.g., –OH, Si–O, C–O, and Al–O) during different hydration periods. Figure 11a, b displays the patterns of L20, L40, and L50 mixes at 28 and 90 days. It is observed that the bands around 798, 778, 695, and 465 cm^−1^ indicate that all samples are quartz-rich, due to the Si–O symmetric stretching band. The quartz band at 445–465 cm^−1^ decreases over curing time as the pozzolanic reaction advances^3^. The Si–O stretching band appears in hydrated products like ettringite (or monosulfoaluminate) and CSH, observed at 1008–1023 cm^−1^, and increases with curing time due to ongoing hydration and the formation of more hydration products^8^. The band at 1430 cm^−1^ may be linked to different CO_3_^−2^ environments, such as calcium carboaluminate (CC), which increases with curing time as a result of carbonation, evident at 90 days. For L20, an increase in the 780 cm^−1^ band indicates a growing amount of unreacted silica. In cementitious materials containing calcined clay, silica is a key component for the pozzolanic reaction, where it reacts with calcium hydroxide (CH) to form additional calcium-silicate-hydrate (C–S–H) gel^43^. The increasing intensity of this band over time (28 to 90 days) suggests that the pozzolanic reaction slows down or has become incomplete. The presence of unreacted silica, instead of forming a strong C–S–H gel, weakens the overall matrix. A less dense and less integrated microstructure will have reduced compressive strength and overall mechanical performance^44^. Furthermore, the increase of the absorption band of CO_3_^−2^ at 90 days indicates progressive carbonation, which leads to weaker and more porous microstructures, as CO_2_ diffusion occurs more easily, thereby accelerating deterioration and further reducing mechanical performance in L20 as noted in Fig. 8^45^.

### Characterization of colored render mortars based on CCL-HL binder

Lime mortars have been thoroughly researched and developed over time. Commonly, additives like pozzolans are used to enhance both the workability and strength of these mortars. This study aims to evaluate the potential of substituting hydrated lime in lime mortars with calcined clay. Based on the findings from the previous section (see “CCL-HL as binder in mortars” section) and the mechanical results from various CCL-HL mortar mixes, the optimal mix ratio for achieving the best mechanical performance is the L40 mix. The focus of this part of the study is to utilize this mix (L40) to create different colored render mortar mixtures and to examine their mechanical and physical properties.

#### Workability, water demand, setting time, and flow value

The results presented in Table 5 indicate the water–solid ratio, setting time, and flow value of the prepared colored render mortars. It was observed that as the binder content (L40) increases and the sand content decreases, the water–solid ratio also increases, resulting in a reduction in setting time. Additionally, higher amounts of inorganic pigment require more water, which subsequently increases the setting time. Moreover, the workability of the render mortars decreases with an increase in L40 content, leading to lower flow values as per EN1015-3. In contrast, higher sand content enhances workability. Among the tested mixes, R25 showed the best workability and flow rate when compared to the reference blank sample for both types of inorganic pigments (as shown in Table 5).

**Table 5:** Water-to-solid ratio, setting time, and flow values of colored render mortars using L40 binder.

Mix. no	W/S (%)	Setting time (min.)		Flow value (mm)
		I.T	F.T	
Blank	26.00	320.00	520.00	165.00
R25G	22.70	510.00	680.00	182.00
R30G	27.60	480.00	620.00	176.00
R35G	30.50	450.00	570.00	170.00
R25Y	22.20	490.00	660.00	177.00
R30Y	26.80	460.00	590.00	170.00
R35Y	29.60	410.00	530.00	164.00

The addition of HPMC as a water-retaining agent improves workability. However, it is crucial to use it in appropriate amounts, as an excessive dosage can lead to issues such as segregation and sagging^46^. Additionally, it has been observed that rendering mortars containing yellow pigment have a lower flow value compared to those with green pigment. This difference is likely due to the needle-shaped particles found in the yellow pigment^23^.

#### Mechanical properties of the prepared colored render mortars

**Fig. 12:**
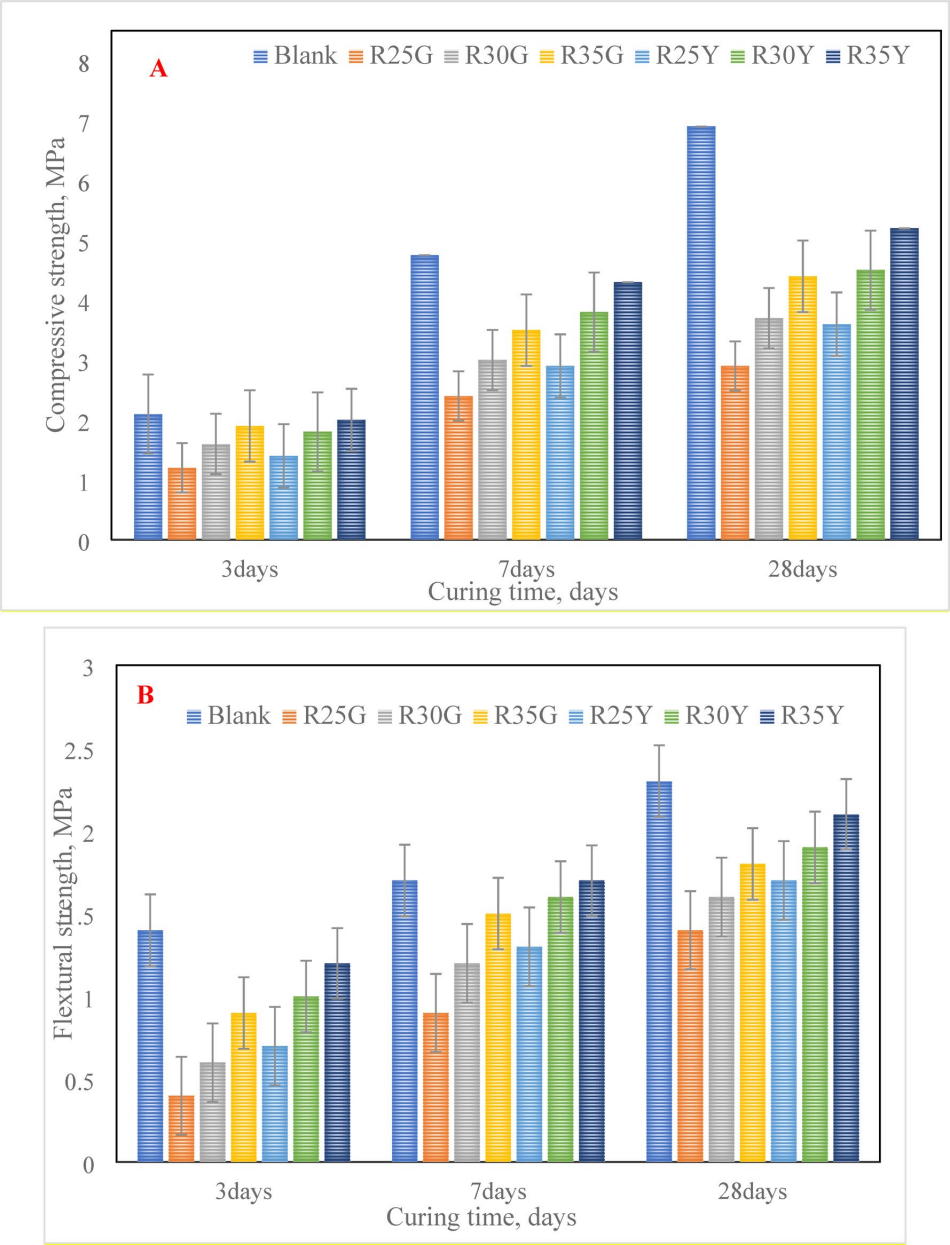
The mechanical characteristics of different colored render mortars based on L40 binder up to 28 days: (**a**) Compressive strength, (**b**) Flexural strength.

The compressive and flexural strengths of the prepared render mortars (blank, R25G, R30G, R35G, R25Y, R30Y, and R35Y) are shown in Fig. 12a, b. The results demonstrate that both compressive and flexural strengths increase with curing time, reaching their maximum at 28 days. This improvement is due to ongoing hydration processes that produce hydration products like stratlingite, which enhances the mechanical strength of the CCL-HL binder. Strength gains are observed as the curing period extends and the CCL-HL content increases, leading to a denser microstructure^37^. The addition of hydroxypropyl methylcellulose improves the cohesion of the render mortars by preventing water from draining away^47^. It was also observed that the mechanical strength of the render mortars (R25G, R30G, R35G, R25Y, R30Y, and R35Y) decreases when the L40 content is reduced. Generally, increasing the binder (CCL-HL) content boosts mortar strength, but too much binder can cause significant shrinkage and cracking. To prevent cracking, the mortar should have high tensile strength and a low modulus of elasticity, enabling it to better withstand applied stresses^48,49^.

Typically, adding a polymer binder (L40) offers several benefits, including better water retention, improved adhesion, higher flexural strength, reduced permeability, and enhanced workability^31^. All the prepared colored render mixes were suitable for use as render mortars, satisfying the requirements of EN998-1/2010. However, the results showed that the compressive strength of all colored mortars (R25G, R30G, R35G, R25Y, R30Y, and R35Y) was lower than that of the blank sample. This is because the hydration products formed in the blank sample, such as calcium silicate hydrate (CSH) and calcium aluminate hydrate (CAH), are denser than those in the other mortars.

The CSH produced from the pozzolanic reaction has a lower density compared to that formed from the hydration of the blank. Additionally, including about 15% limestone in the render mortars reacted with alumina to form a carboaluminate phase, which precipitated in the voids and contributed to increased compressive strength. This strength was further improved by the pozzolanic reaction between HL and CCL^48,49^. Variations in the hydration processes of the L40 binder, especially those involving pozzolans, likely explain the differences in strength^50^. The results for the R25G, R30G, R35G, R25Y, R30Y, and R35Y mixes aligned with expectations: as the proportion of yellow and green pigments increased, so did the compressive strength. This suggests that the pigment color used in the mix directly influences the material’s strength. Nonetheless, the pigment ratio in the colored mortars should be kept below 6%^51^.

#### Comparison of various characteristics between eco-friendly CCL-HL colored render mortar (R35Y) and traditional cement render mortar (blank)

**Fig. 13:**
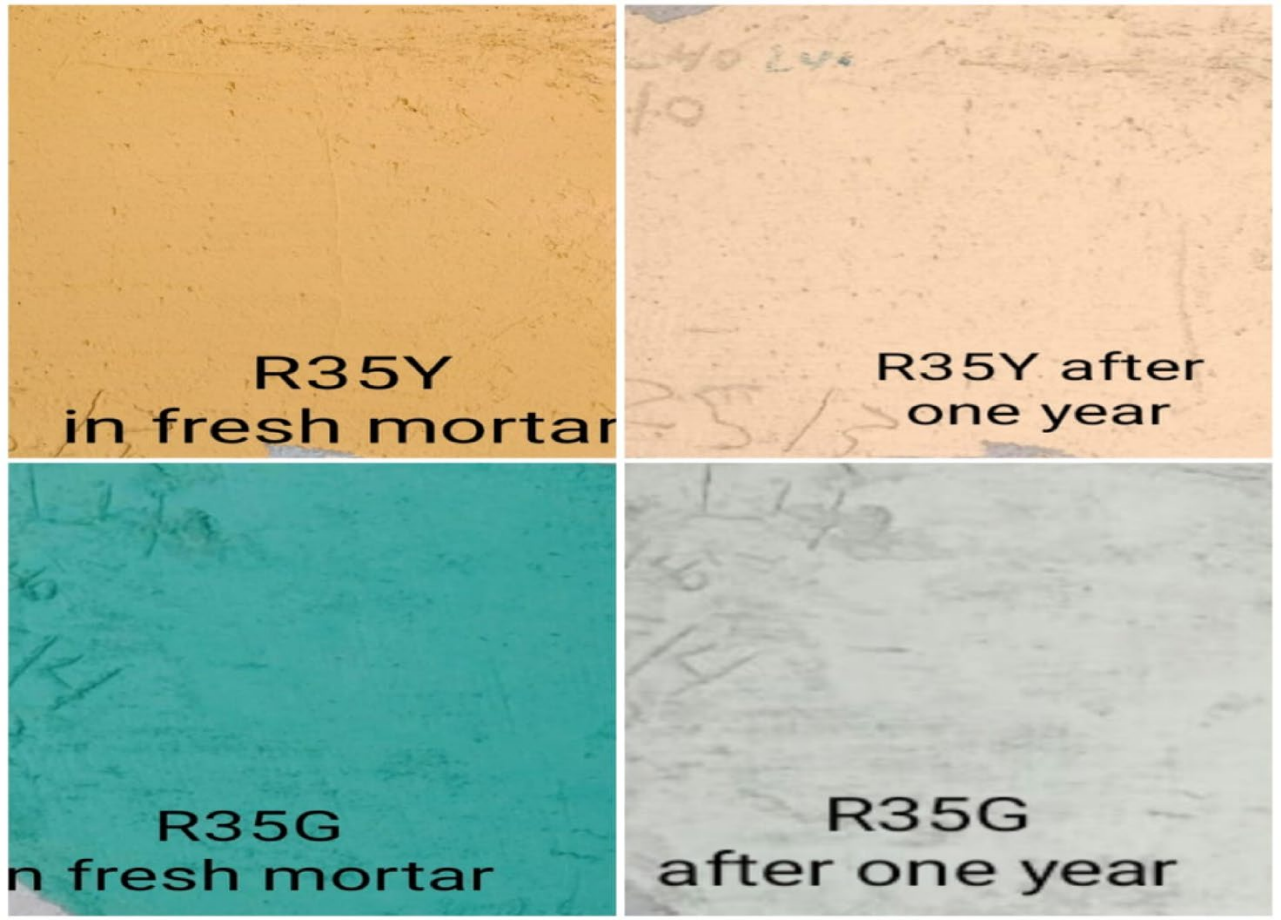
The visual appearance of colored render mortars incorporating green and yellow pigments in fresh mortar and after 1 year on the substrate.

Selecting suitable colored rendering mortars requires a careful balance between performance, cost, and environmental impact. In the second phase of this study, the R35Y mix was evaluated as a potential-colored render mortar by comparing its microstructural properties, porosity, bulk density, mechanical adhesion, and thermal conductivity to those of a plain cement render mortar. Although the R35G mix showed similar mechanical performance to the R35Y, it has several drawbacks. Its higher cost and the fact that its green color fades over time to a lighter shade make it less desirable (Fig. 13). The R35G mix also has lower mechanical strength than the R35Y (Fig. 12a, b), despite both mixes containing no more than 6% inorganic pigment^23,24^. This is a significant concern, especially in contexts where cost efficiency and sustainability are crucial.

The study also highlighted the practical advantages of the R35Y mix, as yellow ferric oxide powder, the inorganic pigment in its formulation, is more readily available than green chromium oxide. This accessibility helps explain the popularity of yellow render mortars in regions like Egypt, demonstrating how material availability influences construction choices. In summary, the R35Y mix offers a promising alternative, providing a balance of acceptable performance, cost-effectiveness, and a reduced environmental impact, supported by the easy availability of its primary pigment component.

**Fig. 14:**
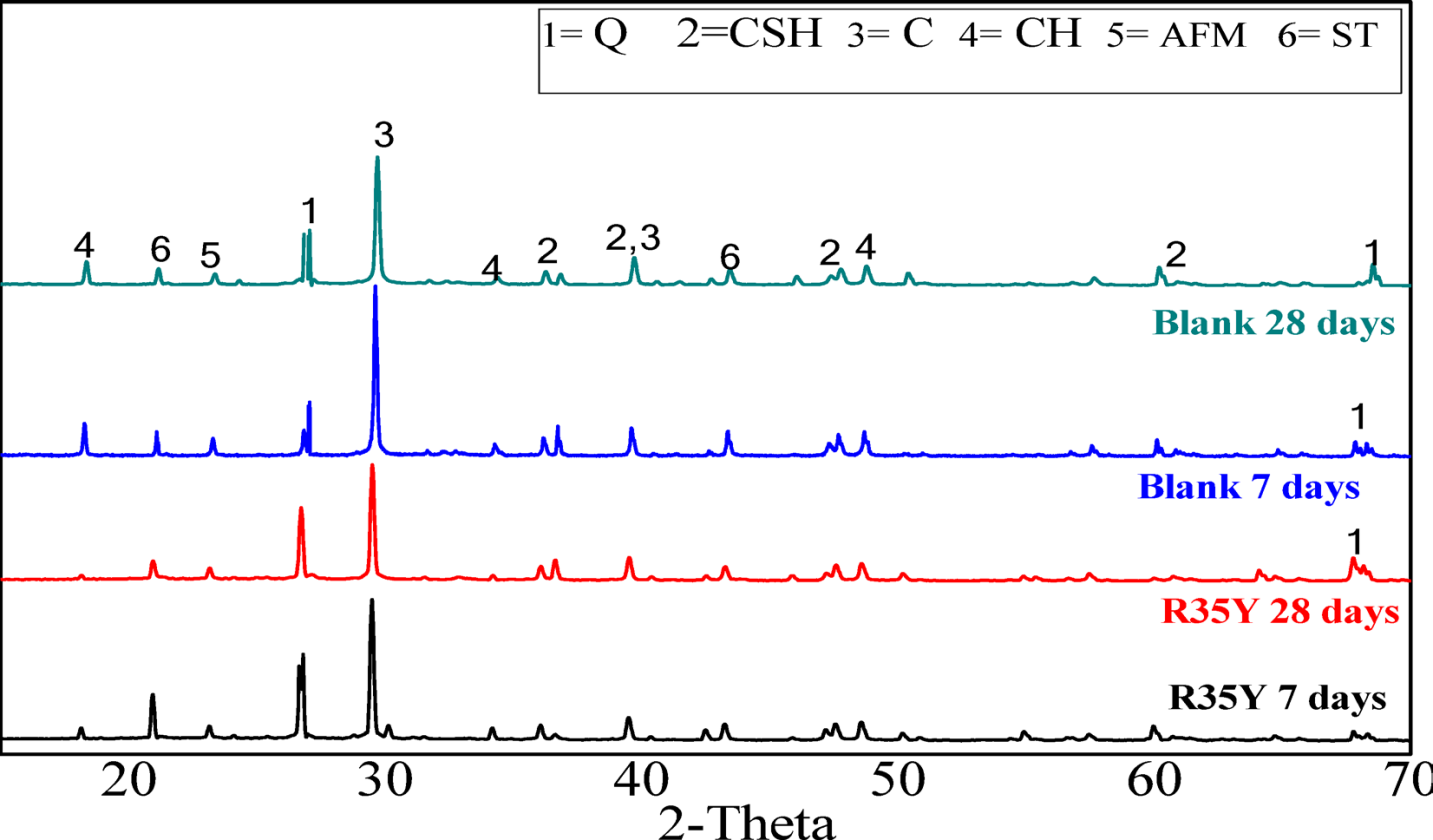
XRD patterns of colored render mortar based on LH-CCL binder (R35Y), and blank mortar sample at 7 and 28 days (Q = quartz, CSH = calcium silicate hydrate, C = calcite, CH = portlandite, AFM = moncarboaluminat. ST = stratlingite).

*XRD of hardened render mortars* The X-ray diffraction (XRD) analysis was conducted on the selected mixes of render mortar for sample R35Y, which was based on CCL-HL as the binder. This analysis also included a blank mortar mix, as illustrated in Fig. 14. The analysis identified several main phases, including:

- C_2_ASH_8_, with d-values of 4.52, 2.59, and 2.34 Å.
- Calcium silicate hydrate (CSH), with d-values of 3.61, 3.08, 2.56, and 1.76 Å.
- Monocarbonate (AFm), with d-values of 8.43 and 2.69 Å.
- Portlandite (CH), with d-values of 5.23, 3.39, and 2.66 Å.
- Quartz (Q), with d-values of 4.59 and 2.78 Å.
- Calcite, resulting from carbonation and found in the limestone content of the mixes, with d-values of 9.72 and 3.11 Å.

Calcium carboaluminate primarily forms through the reaction of calcium carbonate with the C₃A phase. As depicted in Fig. 14, quartz (d = 4.59 Å) is the main component of unreacted CCL, which is a significant component of the R35Y render mortar mix. CCL can react with limestone to produce mono- and hemi-calcium aluminate, leading to a refined pore structure^52^. Furthermore, the presence of limestone suppresses the formation of strätlingite, as a larger portion of aluminum is utilized in the formation of monocarbonate and hemicarbonate AFm phases (d = 8.43 and 2.69 Å), while retaining ettringite^52^.

**Fig. 15:**
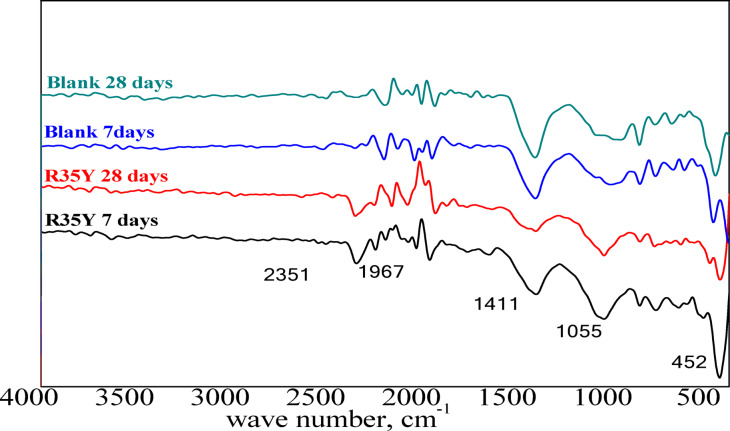
FTIR patterns of render mortar based on CCL-HL binder and blank mortar sample at 7, 28 days.

*Fourier transform infrared spectroscopy (FTIR)* FTIR spectroscopy characterizes changes in the environments of functional groups (e.g., –OH, Si–O, C–O, and Al–O) in CCL-HL render mortars and their hydration products under moderate temperature treatments. Figure 15 shows key bands for render mortar mix R33Y compared to a blank mortar mix. The absorption band at approximately 1050 cm^−1^, along with peaks at 875 cm^−1^ and 755 cm^−1^, is attributed to the Si–O asymmetric stretching vibration. Shoulder peaks at 1419 cm^−1^ and 2350 cm^−1^ may be associated with various CO_3_^2−^ environments, including calcium carboaluminate (CC). The Si–O stretching band, present in ettringite or mono-sulfoaluminate^8^ and observed at 1062 cm^−1^, decreases with curing time, indicating the progress of hydration^8^.

*The bulk density of hardened render mortars* The bulk density of selected hardened render mortars based on CCL-HL (R35Y) and a blank sample up to 28 days is presented in Table 6. The results indicate that the bulk density of the blank render mortar gradually increases with curing time up to 28 days. This increase may be attributed to the continuous formation of hydration products, which fill some of the pores, thereby increasing the bulk density of the hardened render mortar. For the R35Y sample, the bulk density rises initially but decreases by 28 days. This decrease can be explained by the under-dry conditions, where moisture evaporates. According to the formula for bulk density (mass/volume or M/V), a reduction in mass results in a decrease in bulk density^53^. Conversely, under wet conditions, the opposite occurs. Furthermore, the bulk density of the R35Y sample is higher than that of the blank sample. This may be because in mortars composed of HL and CCL, higher water content generally leads to increased initial bulk density, but this density tends to decrease with longer dry curing times^54^.

**Table 6:** The bulk density of hardened R35Y and blank mortar samples up to 28 days.

Sample number	Bulk density (g/cm^3^)		
	3 days	7 days	28 days
Blank	2.532	2.605	2.612
R35Y	2.912	3.116	2.918

**Fig. 16:**
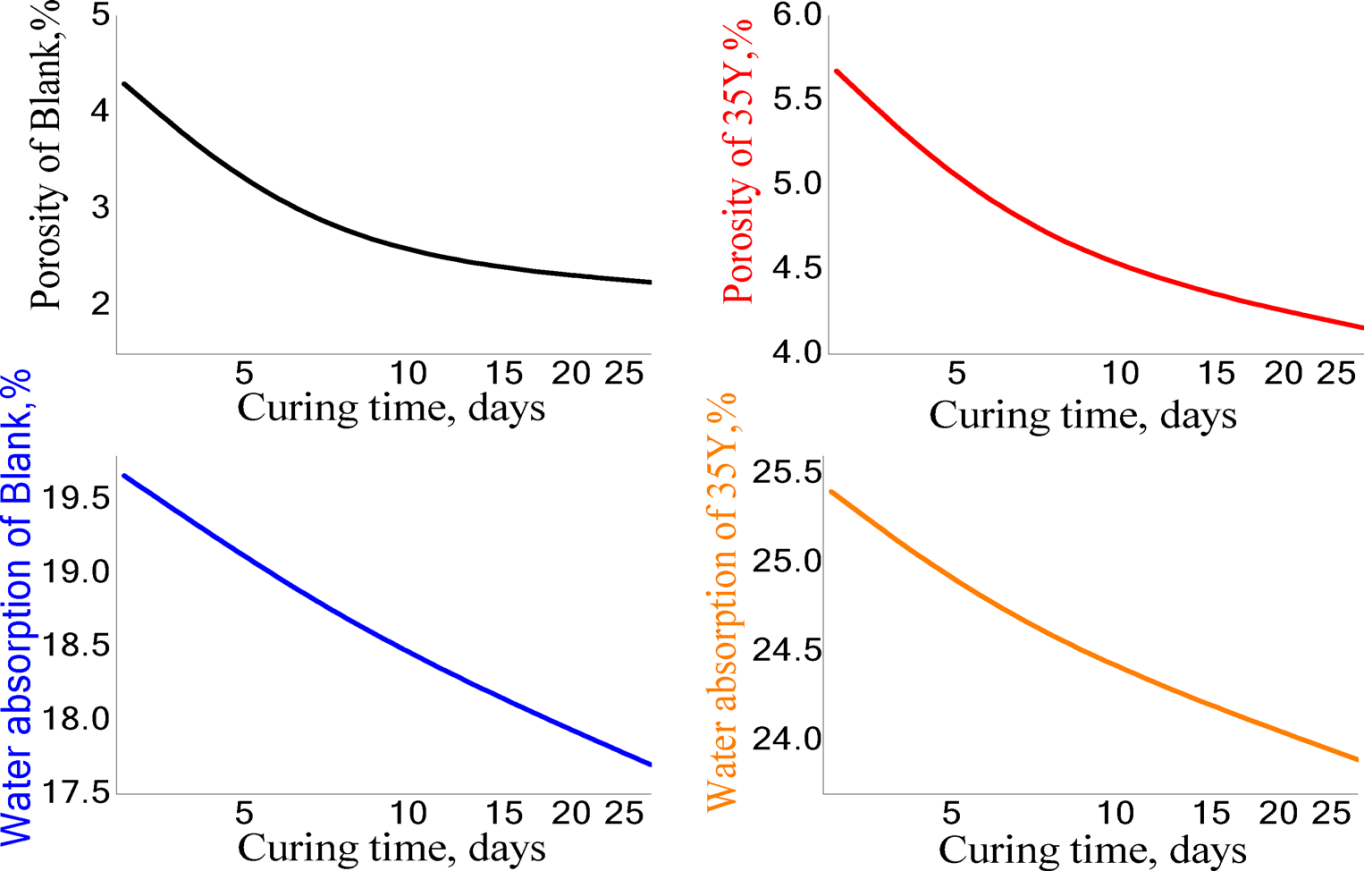
The porosity and water absorption of the R35Y and blank render mortars 28 days of hydration.

*The porosity and water absorption of render mortars* The porosity and water absorption of hardened render mortar (R35Y) and the blank sample after 28 days are illustrated in Fig. 16. The results indicate that the porosity and water absorption of both samples decrease over time as curing progresses. This reduction in porosity is attributed to the ongoing formation of additional hydration products, which fill the pores. As a result, pore connectivity diminishes, and the material becomes more compact, ultimately enhancing its strength. Moreover, hydration reactions can continue for an extended period, leading to lower overall porosity and the development of finer pore structures^55–58^.This advancement in hydration also contributes to a decrease in carbonation levels, as shown in Figs. 14 and 15. These figures reveal a reduction in the carbonate group bands observed in FTIR analyses and a decrease in calcite peaks in XRD analyses of the mortar samples over time. Such changes indicate a transition towards a more stable and durable material, thereby improving its resistance to environmental factors. Overall, the results suggest a decline in porosity along with an increase in compressive strength of the mortars over time. Notably, the porosity and water absorption of the R35Y sample were higher than those of the blank mix, which is consistent with the compressive strength results. This disparity may be due to the lower hardened bulk density of the reference sample, as lightweight mortars typically have a higher volume^59^. Additionally, the high water-to-cement (w/c) ratio used in the manufacturing process may also play a role^60^. In an air-cured environment, water gradually evaporates, causing the mortars to shrink and the water-filled gaps to close. Hydration continues only with the water initially present in the mix and stops once this water is no longer available. Consequently, as hydration progresses and water is lost, both porosity and water absorption decrease over time^47,48^.

#### Adherence to the Substrate (pull off) and thermal conductivity

The study of the mechanical adherence of the render, based on the substrate’s suction, is summarized in Table 7. The findings indicate that the pull-off render mortars based on CCL-HL (R35Y) do not perform better than the blank sample. This could be attributed to the blank sample’s higher fine filler content, which may result in increased adherence to the substrate. Moreover, a richer binder content in the mortars may lead to more cracking, potentially causing a loss of adherence^61^. The R35Y mix has greater porosity, which enhances absorption by the substrates. In contrast, the blank mix has low porosity with excessive absorption capacity, leading to rapid desiccation of the coating. This hinders the hydration of its components and results in lower adherence. Mortars with higher water absorption levels tend to reduce the render’s adhesion^62^.

**Table 7:** The pull-off and thermal conductivity of render mortar R33Y and blank mortar samples.

Sample No	Pull off (N/mm^2^)	Thermal conductivity (W/m k)
Blank	0.20	0.52
R35Y	0.32	0.46

On the other hand, Table 6 presents the results from the thermal conductivity test. The heat conduction in materials is directly linked to their microstructural and compositional characteristics, as the conduction mechanism involves both vibrational waves and electron movement. The results reveal that the thermal conductivity of R35Y is lower than that of the blank sample, likely due to its higher porosity, which makes it lighter and increases its pore volume fraction. As a result, the heat conduction mechanism is altered. Generally, a higher conductivity coefficient indicates lower porosity and better compactness but is associated with a weaker thermal insulation effect^24^.

In terms of thermal performance in buildings, porous materials offer advantages for thermal insulation due to their low conductivity^63^. The type of material used can significantly impact the thermal performance of a building, with porous materials being particularly beneficial for thermal insulation because of their reduced conductivity^63^.

#### The limitations of the present study and future work

This study successfully demonstrates the feasibility of calcined clay as a substitute for hydrated lime in lime-calcined clay binders used in colored render mortars, particularly regarding early-age mechanical and microstructural properties. However, the research is limited by the absence of long-term durability assessments, specifically concerning sulfate attack and freeze–thaw resistance. Additionally, shrinkage behavior was not evaluated. Future research should prioritize durability testing under varying environmental conditions and investigate the compatibility of these mortars with diverse substrates to provide a more comprehensive understanding of their performance.

## Conclusions

This research investigates the feasibility of using calcined clay, thermally activated at 750 °C, as a substitute for hydrated lime in a hydrated lime-calcined clay binder. The aim is to lower production costs and reduce energy use associated with cement manufacturing by replacing traditional mortar with this affordable binder. The study explores various proportions of calcined clay (90%, 80%, 70%, 60%, and 50%) within the compositions of the hydrated lime-calcined clay binder. It also assesses the suitability of the lime-calcined clay binder for colored render mortar. Gray clay from Egypt is used, with 1, 1.5, and 2% inorganic pigments added for aesthetic purposes (see Fig. 1). This research emphasizes the mechanical and microstructural properties of the render mortars containing these pigments, evaluating their compliance with EN998-1/2010 standards. The goal is to determine if colored render mortars using a CCL-HL binder are viable for decorative applications. The results highlight several key conclusions:

- The lime-to-pozzolana ratio greatly influences the water demand of CCL-HL mortar. Increasing lime content lowers water demand and setting time because it accelerates the pozzolanic reaction, while higher calcined clay content raises water demand due to the clay’s properties.
- The study shows that replacing 40% of CCL with hydrated lime in CCL-HL binders improves both compressive and flexural strength by enhancing pozzolanic reactions and hydration. However, the potential instability of calcium aluminate hydrate at high lime levels indicates that long-term performance should carefully consider lime usage.
- XRD analysis of CCL-HL mortar mixes confirms the formation of hydrated phases and pozzolanic reactions across all mixes, with the L20 mix showing a decrease in stratlingite and compressive strength at 90 days. Calcium aluminate is detected in all mixes. FTIR analysis reveals that curing CCL-HL mortar mixes at moderate temperatures causes changes in functional groups, confirming the quartz-rich nature, progression of pozzolanic reactions, increased calcium carboaluminate from carbonation, and reduction of ettringite/mono-sulfoaluminate due to hydration.
- The composition of render mortars—specifically the ratios of binder, sand, and pigment—significantly impacts their water–solid ratio, setting time, and flow properties. Additionally, additives such as HPMC and the type of pigment used also affect these characteristics. This highlights the importance of careful mix design to achieve optimal mortar properties.
- All prepared render mortars met EN998-1/2010 standards. The addition of pigment generally enhances compressive strength, while flexural strength is particularly sensitive to increased proportions.
- XRD analysis of R35Y render mortar with 35% L40 binder and 2% yellow pigment identified key hydration products and mineral phases, indicating a pozzolanic reaction that leads to calcium carboaluminate formation and pore structure refinement. Complementary FTIR spectroscopy confirmed changes in functional groups related to hydration, particularly affecting Si–O bonds, further supporting the ongoing hydration process within the mortar.
- The R35Y sample initially experienced an increase in bulk density, but it decreased by day 28. R35Y had a higher bulk density due to increased water content, but it did not show superior mechanical adhesion compared to the blank.
- The R35Y render mortar sample exhibited lower thermal conductivity than the blank sample, owing to its increased porosity and pore volume fraction, highlighting its potential for improved thermal insulation in buildings.

Our research presents a new, eco-friendly, and sustainable alternative to Ordinary Portland Cement (OPC) for use in render mortar. This alternative not only enhances the aesthetics of buildings but also aids in the preservation of older structures. Additionally, it helps reduce the carbon footprint typically associated with traditional cement mortars.

## Data Availability

The authors confirm that the data supporting the findings of this study are available within the article. The datasets analysed during the current study are available from the corresponding author on reasonable request. The authors declare that the data supporting the findings of this study are available within the paper files. Should any raw data files be needed in another format, they are available from the corresponding author upon reasonable request. Source data are provided with this paper.
